# Ligand-binding domains of nuclear receptors facilitate tight control of split CRISPR activity

**DOI:** 10.1038/ncomms12009

**Published:** 2016-07-01

**Authors:** Duy P. Nguyen, Yuichiro Miyaoka, Luke A. Gilbert, Steven J. Mayerl, Brian H. Lee, Jonathan S. Weissman, Bruce R. Conklin, James A. Wells

**Affiliations:** 1 Department of Pharmaceutical Chemistry, University of California, San Francisco, San Francisco, California 94158, USA; 2 Gladstone Institute of Cardiovascular Disease, San Francisco, California 94143, USA; 3 Department of Cellular and Molecular Pharmacology, University of California, San Francisco, San Francisco, California 94143, USA; 4 Howard Hughes Medical Institute, University of California, San Francisco, California 94158, USA; 5 Department of Medicine, University of California, San Francisco, San Francisco, California 94143, USA

## Abstract

Cas9-based RNA-guided nuclease (RGN) has emerged to be a versatile method for genome editing due to the ease of construction of RGN reagents to target specific genomic sequences. The ability to control the activity of Cas9 with a high temporal resolution will facilitate tight regulation of genome editing processes for studying the dynamics of transcriptional regulation or epigenetic modifications in complex biological systems. Here we show that fusing ligand-binding domains of nuclear receptors to split Cas9 protein fragments can provide chemical control over split Cas9 activity. The method has allowed us to control Cas9 activity in a tunable manner with no significant background, which has been challenging for other inducible Cas9 constructs. We anticipate that our design will provide opportunities through the use of different ligand-binding domains to enable multiplexed genome regulation of endogenous genes in distinct loci through simultaneous chemical regulation of orthogonal Cas9 variants.

## Abstract (web summary)

CRISPR-Cas9 has been widely used to manipulate genomes, however control over activity is necessary to explore transcriptional or epigenetic regulation. Here the authors use a split Cas9 fused to nuclear receptor ligand-binding domains to achieve tuneable Cas9 activity.

## Graphical abstract

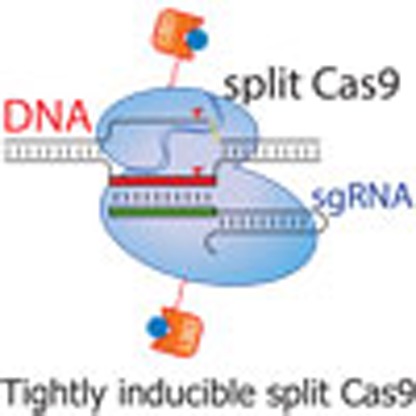

In bacteria and archaea, the clustered, regularly interspaced, short palindromic repeats (CRISPR)-CRISPR-associated (Cas) systems provide adaptive immunity via short mature CRISPR RNAs that guide the system to target invading foreign DNA^1^,^2^. The Cas9 protein from bacterial type II CRISPR-Cas systems has recently been adapted to induce sequence-specific alterations in many genomic contexts including mammalian genomes^3^,^4^,^5^,^6^. In this Cas9-based RNA-guided nuclease (RGN) system, DNA recognition requires a single-guide RNA (sgRNA) that contains the first 20 nucleotides complementary to the target sequence and a protospacer adjacent motif sequence immediately upstream of the target sequence^3^,^5^,^6^,^7^,^8^,^9^. Redesigning the sgRNA to retarget virtually any DNA sequence thus leads to the ease of construction of RGN reagents for genome editing purposes.

Researchers have recently repurposed the catalytically inactive Cas9 protein (dCas9) as a tool for transcriptional regulation of gene expression and epigenetic modification^8^,^9^,^10^,^11^,^12^,^13^,^14^,^15^,^16^,^17^,^18^. Dissecting the dynamics of transcriptional regulation or epigenetic modifications in complex biological systems requires the ability to control the activity of Cas9 with high temporal resolution. Although doxycycline-inducible promoters have been employed to provide transcriptional control of CRISPR activity, these systems typically take more than 2 days to achieve maximum Cas9 activity and thus do not provide the temporal resolution required in many studies of dynamic systems^19^,^20^,^21^,^22^,^23^.

With the recent advent of dCas9-based techniques in transcriptional regulation and epigenetic modifications, we anticipate a need for a robust and rapidly inducible system for simultaneous orthogonal activation of different CRISPR systems to provide dynamic control of distinct loci. Recent efforts have been directed at developing means to directly control Cas9 activity^24^,^25^,^26^,^27^. Nonetheless, these approaches, including inducible dimerization of split Cas9, have shown significant background activity or have employed chemical probes known to inhibit critical cellular pathways^24^,^25^,^28^. Thus, efforts to interrogate cellular dynamics of gene transcription, epigenetic modifications and genome organization would be greatly aided by techniques that are capable of dynamically controlling Cas9/dCas9 variants without background activity.

Here we report the use of ligand-binding domains from nuclear receptors to facilitate the tight chemical control of split CRISPR-Cas9 activity. Many ligand-binding domains from nuclear receptors are known to interact with cytosolic Hsp90 and thus localized in the cytoplasm in the ligand-unbound state. To create a tightly regulated and inducible split Cas9 system, we sequester the split Cas9 in the cytoplasm by fusing each fragment (N-Cas9 and C-Cas9) with the ligand-binding domain ERT from oestrogen receptor (Fig. 1a)^29^,^30^,^31^. By adding the synthetic ligand 4-hydroxytamoxifen (4OHT) to the cells expressing our designed split Cas9, we trigger the release of the protein fragments bound to Hsp90 and induce their nuclear translocation to reconstitute an active RGN complex. Our approach, in which sgRNA and Cas9 fragments are localized in the nucleus and cytoplasm, respectively, enables rapid CRISPR activation with extremely low background activity and high degree of tunability. We extend this approach to the ligand-binding domain of an orthogonal steroid receptor, glucocorticoid receptor α (GR), that is similarly sequestered in the cytosol in the absence of glucocorticoid for independent small-molecule regulation of split CRISPR activity. Fusing ligand-binding domains of different nuclear receptors to orthogonal Cas9 variants^32^,^33^,^34^ may therefore find utility in future studies to enable simultaneous genome regulation of endogenous genes in distinct loci through temporal control of orthogonal variants.

## Results

### Identifying suitable split sites

**Figure 1:**
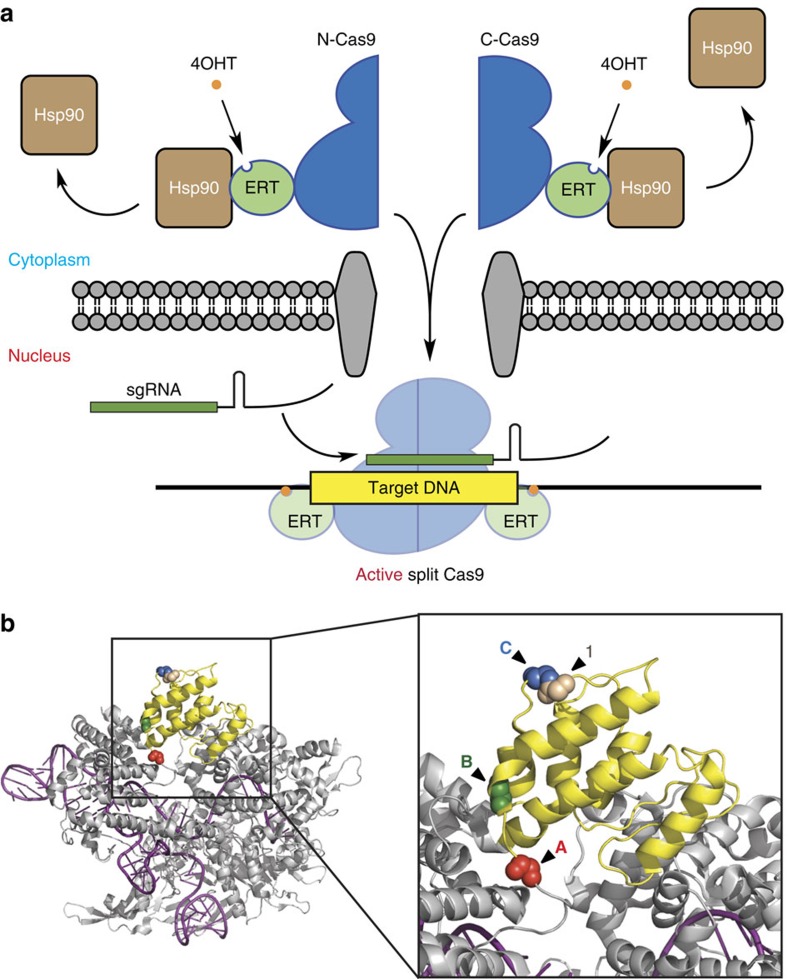
Inducible control of split Cas9. (**a**) Schematic of the strategy for utilizing the ligand-binding domain of the oestrogen receptor, ERT (light green), to achieve tight control of CRISPR activity in split Cas9 (blue). The ERT-fused fragments of split Cas9 are segregated in the cytoplasm from the nuclear-localized sgRNA (green) through their interactions with Hsp90 (brown)^29^,^30^,^31^. Upon addition of 4-hydroxytamoxifen (4OHT, orange), the split fragments are released from Hsp90, and translocate into the nucleus to reconstitute an active split Cas9 complex. (**b**) Locations of split sites for the previously published split-1 (ref. 24; beige) and three split candidates **A** (red), **B** (green) and **C** (blue) in the REC2 domain (yellow) of the Cas9 protein (grey) complexed with sgRNA (purple). PDB 4OO8 (ref. 37).

**Figure 2:**
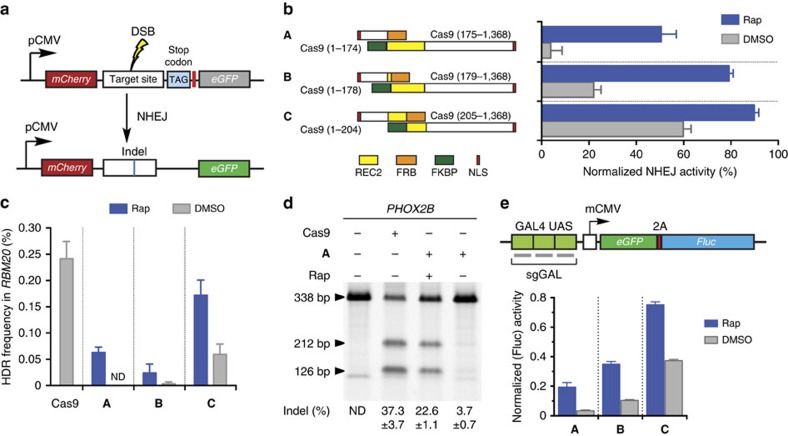
Design and characterization of FRB/FKBP-mediated inducible split Cas9. (**a**) The fluorescence-based NHEJ assay. The fluorescence reporter under CMV promoter consists of an in-frame *mCherry* gene, an amber codon-containing linker sequence and an out-of-frame *eGFP* gene. When Cas9 cleaves the linker sequence between *mCherry* and *eGFP* to produce a double-strand break, the reporter gene is repaired by NHEJ and switches on the EGFP fluorescence. DSB, double-stranded break; pCMV, human cytomegalovirus immediate early promoter. (**b**) The gain-of-fluorescence activities of split Cas9 candidates. The activities were determined by flow analysis to measure the increase in the GFP-positive population in mCherry-expressing HEK293T cells (*n*=3 biological replicates). Values are background-substracted from a negative control (wild-type Cas9 with control sgRNA), and subsequently normalized to a positive control wild-type Cas9 with an sgRNA targeting linker sequence). Rap, rapamycin, was added to 10 nM. (**c**) HDR activities of split candidates at the *RBM20* gene locus. The HDR efficiency was measured by ddPCR assay to determine the frequency of the mutant allele R636S in the wild-type background (*n*=3 biological replicates). ND, not detected. The data are displayed as mean±standard deviation. (**d**) Surveyor assay of split-**A**-mediated indels at *PHOX2B* gene locus. Indel rates are presented as mean±standard deviation for three biological replicates.(**e**) A firely luciferase (Fluc)-based CRISPRa assay. Split candidates **A**, **B**, and **C** of dCas9-VP64 were transiently transfected into a HEK293 reporter cell line stably expressing a Gal4-UAS-GFP-2A-Fluc reporter. One day after transfection, rapamycin was added to induce CRISPRa activity. The luciferase activity was measured 2 days after rapamycin treatment (*n*=3 biological replicates). Values are normalized to a positive control, which is the wild-type dCas9-VP64 with an sgRNA targeting GAL4 sequence, and background-subtracted from a negative control, which is wild-type dCas9-VP64 with a negative sgRNA. mCMV, minimal cytomegalovirus promoter. The data are displayed as mean±standard deviation.

To determine suitable sites for splitting Cas9, we began by selecting sites in the REC2 domain in the recognition lobe of *Streptococcus pyogenes* Cas9 to fuse split fragments with the model dimerization FRB-FKBP system^35^. Since the REC2 domain is structurally and evolutionarily divergent among a large number of Cas9 orthologues^36^,^37^ and contains many flexible loops, we selected three sites in this domain to generate inducible split Cas9 (Fig. 1b). Furthermore, two other sites in this domain were also previously attempted to produce split protein^38^. To test the activity of our split Cas9 proteins, we created a fluorescence-based assay using a reporter similar to the previous system described by Kim and co-workers^6^. In this assay, the out-of-frame *eGFP* gene is downstream of an *mCherry* gene with an in-frame stop codon. Cas9-mediated cleavage of the linker sequence, upstream of stop codon, allows the *eGFP* gene to be back in frame due to imprecise DNA repair via non-homologous end joining (NHEJ), resulting in green fluorescent protein (GFP) fluorescence (Fig. 2a). We tested our designed split Cas9 combinations by expressing each in HEK293T cells together with the fluorescence reporter and an sgRNA that targeted the linker sequence between *mCherry* and *eGFP* genes. After overnight transfection, cells were treated with 10 nM rapamycin to induce FRB-FKBP dimerization. Three days after induction, cells were harvested and analysed on a flow cytometer for increase in eGFP-positive population.

The gain-of-fluorescence assay revealed that the presence of rapamycin induced the endonuclease activity of Cas9 significantly with all three split Cas9 candidates (Fig. 2b and Supplementary Fig. 1). Among three candidates, split-**A** demonstrates the lowest background activity in the absence of rapamycin while retaining ∼50% of wild-type Cas9 activity, whereas split-**C** has the highest background and induced activity. The site where the Cas9 was split to create split-**C** is adjacent to split-1 recently reported in Zetsche *et al.*^24^ (Fig. 1b), which was also shown to have high background activity.

To evaluate the efficiency of our split Cas9 candidates in stimulating homology-directed repair (HDR), we performed a digital-droplet PCR-based assay to detect the introduction of a single-base mutation R636S in *RBM20* gene via split Cas9 (Fig. 2c and Supplementary Fig. 2)^39^. Combining Taqman PCR and digital droplet PCR (ddPCR), this assay is able to distinguish both wild-type and mutant allele, allowing us to quantify the efficiency of homologous recombination in *RBM20* gene. The ddPCR-based assay revealed a similar trend in rapamycin-induced activity that was observed in the gain-of-fluorescence assay. Significantly, for split-**A**, no background HDR activity was detected, and up to ∼25% of wild-type activity was observed upon the addition of rapamycin. We further used split-**A** to target the *PHOX2B* locus and performed the Surveyor assay to validate the inducible activity of this split candidate (Fig. 2d)^39^. The Surveyor assay showed high insertion–deletion (indel) rate (22.6%) in induced cells. However, without addition of rapamycin, cells transfected with split-**A** showed only minimal indel rate (∼3%), confirming the low background activity of split-**A** observed in our previous endonuclease assays.

To further assess the leakiness of our designed split Cas9, we used the previously described luciferase gene reporter^9^ to evaluate the ability of our split candidates to bind specific DNA sequences, instead of detecting its endonuclease activity. Several laboratories previously reported the use of the CRISPR activator (CRISPRa) system in which recruitment of a catalytically inactive Cas9 mutant (dCas9) fused with a transactivation domain, such as VP64, to upstream sequences of a specific gene is able to induce transcriptional activation^9^,^11^,^12^,^13^. Thus, we assessed the ability of our split candidates to activate the expression of the GFP-luciferase gene reporter (Fig. 2e). Since there are three binding sites in the GAL4 UAS sequence to which dCas9 can bind to, we reasoned that this luciferase-based CRISPRa assay should provide increased sensitivity to detect any leakiness of the inducible split Cas9.

We observed a similar trend, with split-**A** displaying the lowest level of activity in the absence of rapamycin (Fig. 2e). However, split-**A** still retains significantly higher background activity, ∼3% of full-length dCas9-VP64, than wild-type Cas9 co-expressed with negative sgRNA control. This is in good agreement with several previous reports in which sgRNA was shown to act as a constitutive dimerizer for split Cas9 fragments and thus contributes to the background activity in the absence of rapamycin^24^,^28^.

### ERT facilitates tight control of split CRISPR activity

**Figure 3:**
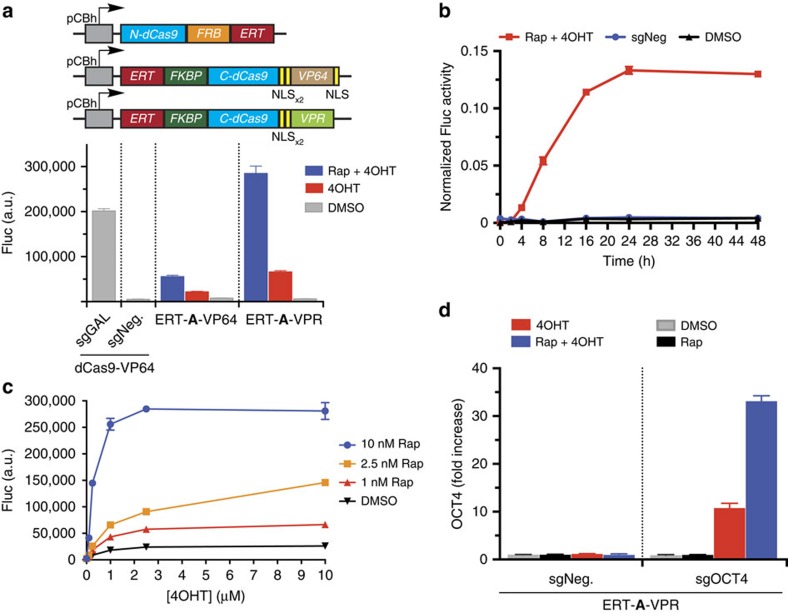
Tight control of inducible split Cas9 with ligand-binding domain ERT. (**a**) Inducible split dCas9 was optimized by fusing ERT to each split fragment to create a split dCas9 responsive to rapamycin (Rap) and 4-hydroxytamoxifen (4OHT). In the N-terminal fragment, the ERT domain is fused to the C-terminus following the FRB domain. Vice versa, the ERT domain is fused to the N-terminus of the C-terminal fragment followed by the FKBP domain. The activation domain used for CRISPRa is either VP64 or VPR domain. The luciferase reporter cells were transfected with plasmids expressing either ERT-**A**-VP64 or ERT-**A**-VPR, and induced with 4OHT (10 μM) and/or Rap (10 nM). The data are displayed as mean±standard deviation of the luciferase activity for three biological replicates. pCBh, chicken β-actin promoter. (**b**) Luciferase activity of cells expressing ERT-**A**-VPR measured 2, 4, 8, 16, 24 and 48 h after induction with 4OHT (10 μM) and/or Rap (10 nM; *n*=3 biological replicates for each time point). Values are normalized to a positive control, which is the wild-type dCas9-VPR with an sgRNA-targeting GAL4 sequence, and background-subtracted from a negative control, which is wild-type dCas9-VPR with a negative sgRNA. The data are presented as mean±standard deviation. (**c**) The optimized inducible split ERT-**A**-VPR facilitates tunable transcriptional activation of reporter gene. The luciferase reporter cells were transfected with plasmids expressing ERT-**A**-VPR, and induced with various concentrations of 4OHT and Rap (*n*=3 biological replicates). The data are shown as mean±standard deviation. (**d**) ERT-**A**-VPR mediates inducible transcriptional activation of endogenous Oct4 expression in HEK293T cells, measured by RT–qPCR. Two previously validated sgRNAs (sgPOU5F1-3 and sgPOU5F1-5) were expressed to target upstream of the transcription start site^19^. Cells were induced with 4OHT (10 μM) and/or Rap (10 nM). Fold-increase values are mRNA expression levels (mean±standard deviation) relative to a negative control sgRNA.

Although the split-**A** has a minimal background activity that is sufficient for Cas9-endonuclease-mediated genome engineering, its leakiness is not optimal for many other CRISPR applications using catalytically inactive dCas9. To further reduce this background activity, we chose to segregate the split Cas9 from sgRNA by sequestering Cas9 in the cytoplasm. To accomplish this, we fused the ligand-binding domain of oestrogen receptor ERT to each split fragments of split-**A** to create a new split dCas9 pair, ERT-**A**-VP64 (Fig. 3a)^31^. Since the ERT domain is sequestered in the cytoplasm by Hsp90 (refs 29, 30, 31) and the sgRNA expressed under U6 promoter is localized in the nucleus^40^, fusing this domain to the Cas9 split fragments should facilitate the tight control of Cas9 activity and eliminate the background caused by sgRNA-mediated dimerization.

Indeed, no significant difference was observed in the luciferase activity between non-induced cells expressing ERT-**A**-VP64 and cells expressing dCas9-VP64 and negative sgRNA, indicating that the split fragments were localized to the cytoplasm and did not produce any nuclear activity (Fig. 3a). Addition of 4OHT, an inducible ligand for ERT, however, allows the split ERT-**A**-VP64 to translocate to the nucleus, resulting in moderate activation of luciferase gene expression. Further enhancement in gene expression to ∼25% of full-length dCas9-VP64 was achieved when both rapamycin and 4OHT were added (Fig. 3a).

To further increase the activity of our split design, we also replaced VP64 with the recently reported activation domain VPR to create ERT-**A**-VPR^15^. Fusing our split construct with VPR allowed the expression of luciferase in the presence of only 4OHT to reach the same level in cells transfected with ERT-**A**-VP64 in the presence of both rapamycin and 4OHT (Fig. 3a). Moreover, upon addition of rapamycin and 4OHT, cells transfected with ERT-**A**-VPR exhibited higher luciferase activity than dCas9-VP64, and achieved a sixfold increase in the activity compared with cells transfected with ERT-**A**-VP64. Fluorescence microscopy also confirmed that eGFP expression is activated on the addition of 4OHT and rapamycin (Supplementary Fig. 3). Immunofluorescent staining against epitope tags fused to each protein fragment confirmed that the majority of both fragments were indeed localized in the cytoplasm and a fraction of protein fragments translocated into the nucleus upon ligand addition (Supplementary Fig. 4a). We thus used the split construct ERT-**A**-VPR for our subsequent time-course- and dosage-dependent experiments.

The time-course measurement of luciferase activity revealed that significant increase in luciferase activity began to be observed 4 h after treatment with rapamycin and 4OHT (Fig. 3b), suggesting a rapid induction response with our inducible system. Furthermore, the activity was observed to reach maximum 24 h after induction. To investigate whether our inducible system is tunable, we tested the activity of our split ERT-**A**-VPR in various concentrations of rapamycin and 4OHT (Fig. 3c). Our dosage-dependent experiment suggests that maximum activation can be achieved at 10 nM rapamycin and 2.5 μM 4OHT. In addition, increasing levels of luciferase activity were observed upon treatment at increasing concentrations of both rapamycin and 4OHT, confirming that our system is highly tunable and can be adapted for applications that require finely controlled gene expression.

We also tested this approach with full-length dCas9-VPR to create a tamoxifen-inducible Cas9. However, dCas9-VPR fused with ERT (ERT-dCas9-VPR) did not show any significant induced activity while retaining the high background observed in our initial split system (Supplementary Fig. 5). Immunofluorescent staining also showed that ERT-dCas9-VPR was not excluded from the nuclei of non-induced cells even though the majority of the protein underwent nuclear translocation upon induction (Supplementary Fig. 5a). The high background could be attributed to the nuclear localization signal (NLS) sequences, which may have prevented ERT domain from fully sequestering the Cas9 protein in the cytoplasm. Removing NLS sequences in both ERT-dCas9-VPR and ERT-**A**-VPR constructs, however, significantly abolished the transcriptional activities (Supplementary Figs 4b and 5b), suggesting that the presence of NLS sequences is required for efficient nuclear translocation of Cas9 constructs. Indeed, the ERT-dCas9-VPR protein lacking NLS sequences remained cytoplasmic in induced cells (Supplementary Fig. 5a). Since sequestration is likely to be incomplete in the presence of NLS, background activity was not observed with the ERT-**A**-VPR construct possibly because both split fragments are required to translocate to the nucleus at sufficient concentration for reconstitution of active Cas9.

To validate that our approach is translatable to temporal control of Cas9 endonuclease activity, we fused ERT domain to each fragment of split-**A** to create a tamoxifen-inducible split Cas9 pair, ERT-**A**. We next performed the Surveyor assay on the endogeneous gene locus *PHOX2B* to verify the inducibility of Cas9 with addition of 4OHT and/or rapamycin (Supplementary Fig. 6a). As expected, no Cas9 activity was detected in the absence of both inducers, confirming the tight control of CRISPR activity through the use of the ERT domain. Furthermore, the Surveyor assay revealed a moderate level of indel rate (∼5%) in *PHOX2B* locus when cells were treated with only 4OHT. In addition, the ddPCR-based HDR assay confirmed the ability of ERT-**A** to induce high HDR activity in RBM20 locus with no background (Supplementary Fig. 6b).

We then sought to determine whether our split design is able to facilitate inducible transcriptional activation of endogenous genes. We tested ERT-**A**-VPR by targeting *POU5F1* (Oct4) for transcriptional activation in HEK 293T cells, using two previously validated sgRNAs that target upstream of the transcription start site^19^. An 11-fold increase in the mRNA level was observed when cells co-expressing ERT-**A**-VPR and *POU5F1*-targeting sgRNAs were treated with 4OHT (Fig. 3d). Furthermore, no increase in mRNA level was observed upon addition of rapamycin, indicating that the presence of 4OHT is required for the activation of our designed split protein. When both rapamycin and 4OHT were added, the mRNA level was further increased to 33-fold, comparable to the transcriptional level observed with the previously reported systems^10^,^17^ (Fig. 3d and Supplementary Fig. 7). This result suggests that our inducible split design is highly functional for temporal control of transcriptional regulation in endogenous genes.

### Optimization of inducible split CRISPR

**Figure 4:**
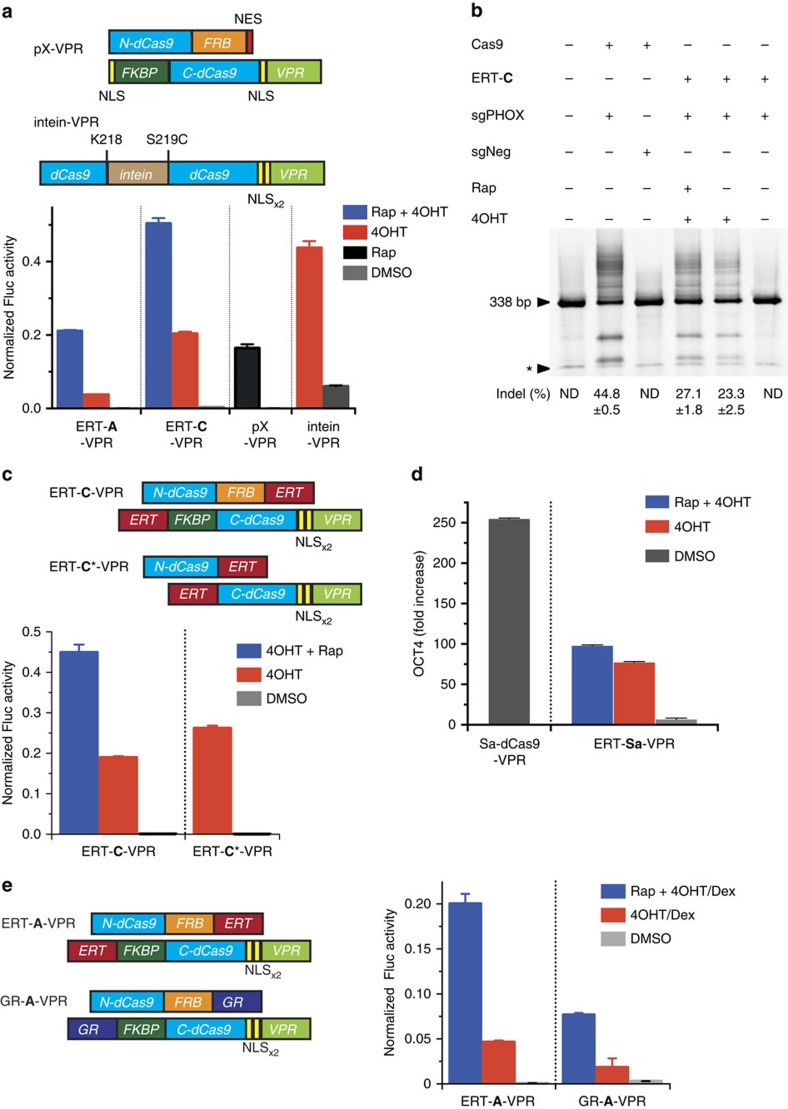
Construct optimization enhances inducible activation of split CRISPR. (**a**) Comparison of split CRISPRa-mediated activity against two previously published systems^24^,^27^. The VP64 domain in a recently reported rapamycin-inducible system (pX855/pX856)^24^ was replaced with the VPR domain to create pX-VPR. The 4-OHT-inducible intein domain^27^ was inserted into the K218/S219 site of the full-length dCas9-VPR construct, which also contains the S219C mutation for protein splicing, to create intein-VPR. The luciferase reporter cells were transfected with plasmids expressing either ERT-**A**-VPR, ERT-**C**-VPR, pX-VPR or intein-VPR and induced with 4OHT (10 μM) and/or Rap (10 nM). The data are displayed as mean±standard deviation of the luciferase activity for three biological replicates. (**b**) Surveyor assay of indels mediated by the optimized inducible split Cas9, ERT-**C**, at *PHOX2B* gene locus. Indel rates are presented as means±standard deviation for three biological replicates. ND, not detected. Asterisk (*) denotes a non-specifically amplified DNA band that is excluded from indel analyses. (**c**) Luciferase activity of reporter cells expressing ERT-**C**-VPR or ERT-C*-VPR, which does not contain the FRB/FKBP domains (*n*=3 biological replicates). The luciferase activity is presented as mean±standard deviation for three biological replicates. (**d**) An inducible split CRISPR system derived from *Staphylococcus areus* Cas9 variant^25^. One sgRNA (sg-Sa-POU5F1) was expressed to target upstream of the transcription start site. Transcriptional activation of endogenous Oct4 expression was mediated by ERT-**Sa**-VPR in HEK293T cells induced with 4OHT (10 μM) and/or Rap (10 nM). The mRNA expression levels were measured by RT–qPCR for three biological replicates 48 h after induction. Fold-increase values are presented as mean±standard deviation relative to a negative control sgRNA. (**e**) Comparison in luciferase activity between cells expressing ERT-**A**-VPR and GR-**A**-VPR (*n*=3 biological replicates). The luciferase reporter cells expressing either ERT-**A**-VPR or GR-**A**-VPR were induced with either 4OHT (10 μM) or Dex (dexamethasone, 10 μM), and/or Rap (10 nM). The normalized activities are presented as mean±standard deviation.

We next extended our approach to our previous split-**C** design, which was shown to have the highest background activity but also retained the highest activity when induced with rapamycin compared with wild-type Cas9 (Fig. 4a). Fusing ERT domains to each split fragments of split-**C** to create ERT-**C**-VPR similarly reduces the background activity significantly while enhancing the transcriptional activity in the luciferase assay by five- to sixfold relatively to ERT-**A**-VPR. When compared with the previously published rapamycin-inducible FRB/FKBP-based CRISPR system^24^, ERT-**C**-VPR exhibited the same level of transcriptional activity in the presence of 4OHT without high background activity observed with the inducible intein system reported by Davis *et al*^27^. In addition, the Surveyor assay on *PHOX2B* locus showed efficient indel formation at ∼23% when cells expressing split-**C**'s tamoxifen-inducible split Cas9 pair, ERT-**C**, were treated with 4OHT (Fig. 4b). Treating the cells with both ligands further increased the indel rate to ∼27%. These indel rates are comparable to the reported rates in previous published systems, suggesting that extending our approach to different split sites could yield more efficient inducible split constructs.

We also investigated whether our split approach can be extended to produce a tamoxifen-inducible system without the use of rapamycin. To achieve this, we removed the FRB/FKBP dimerizing domains from ERT-**C**-VP64 to create ERT-C*-VP64 in which each split fragment is fused only to an ERT domain (Fig. 4c). We reasoned that sgRNA should be sufficient to act as a dimerizer once the nuclear translocation of the fragments is induced with the 4OHT treatment. Indeed, once treated with 4OHT, cells transfected with ERT-C*-VPR exhibited twofold decrease in luciferase activity compared with those transfected with ERT-**C**-VPR and induced with both 4OHT and rapamycin. This result demonstrates that split Cas9 can be solely induced by treatment of 4OHT.

### Generality of inducible split CRISPR design

To demonstrate that a different Cas9 variant can be activated using our approach, we fused FRB/FKBP, ERT and VPR domains to a recently reported split variant from *Staphylococcus areus* Cas9 (SaCas9) to create ERT-**Sa**-VPR for transcriptional activation (Fig. 4d)^25^. We selected this published split site since the REC2 domain is absent in Sa Cas9 and we were unable to identify a suitable split site analogous to those used in our split Cas9 derived from *S. pyogenes* Cas9. Upon addition of both rapamycin and 4OHT in cells expressing ERT-**Sa**-VPR, transcriptional level of endogenous *POU5F1* locus was shown to increase by 97-fold, ∼2.5-fold less than the mRNA level observed in cells expressing **Sa**-dCas9-VPR. Furthermore, treatment of 4OHT sufficiently induces the transcriptional activation close to the same mRNA level observed with both ligands.

We also further explored the use of a known ligand-binding domain from another nuclear receptor, the glucocorticoid receptor α, to create an additional inducible system, GR-**A**-VPR (Fig. 4e). Cells transfected with GR-**A**-VPR exhibited a similar trend in ligand-inducible activation of luciferase expression, despite having a 2.5-fold decrease in transcriptional activity compared with ERT-**A**-VPR. Taken together, these results thus suggest the generality of our approach in using orthogonal ligand-binding domains of different nuclear receptors to achieve tight control of CRISPR activity.

## Discussion

In summary, we have demonstrated here the use of multiple ligand-binding domains from nuclear receptors to create a tightly inducible system of split Cas9. This method enables the temporal control of CRISPR activity in stimulating NHEJ or HDR pathways as well as transcriptional regulation through split dCas9. In contrast to previously published methods, our approach has also allowed us to control Cas9 activity in a tunable manner with a very low background activity. Furthermore, taking advantage of intrinsic dimerization of split Cas9 in the nucleus, we were able to show that only 4OHT is required for ERT-**C**-VPR to trigger dCas9 activity for transcriptional activation. As 4OHT, with its short-term low toxicity^41^, is often used as the agonist of choice in many studies that employ inducible Cre-recombinase for site-specific recombination^42^, we expect that our inducible split CRISPR system will be similarly advantageous in many animal studies. By exploring various split sites or orthogonal analogues^25^,^32^,^34^, we anticipate that 4OHT-induced activity can be further enhanced to obviate the need for rapamycin to fully reconstitute Cas9.

Splitting Cas9 also yields smaller construct size, and thus potentially facilitates more efficient viral packaging in recombinant adenovirus-associated viruses (rAAV)^43^. Both Bao and Ortiz groups recently reported that an intein system could be used to facilitate trans-splicing of split Cas9 and adapted to rAAV-mediated delivery for transgene expression of each split half^44^,^45^. Since the transgene in rAAV vectors tends to suffer from delayed onset of expression^46^,^47^, conditional control will be required to define the temporal window of CRISPR activity. As cell type-specific promoters can be incorporated in rAAV vectors to confer subtissue specificity for transgene expression^48^, our future approach will be directed to optimize the viral constructs to activate CRISPR activity in specific cell type in tissue context.

In addition, the Koh groups and many others reported the development of several orthogonal ligand-nuclear receptor pairs through ‘bump-hole' approach^49^,^50^,^51^. We are thus looking into further extending our split design to these ligand–receptor pairs since the reported ligands do not activate endogenous receptors and constitute cellular toxicity. As shown in our study, we have also successfully demonstrated our approach with the ligand-binding domain of glucocorticoid receptor α to create an analogous inducible system. With many other known nuclear receptors such as thyroid receptor^52^ and non-steroidal ecdysone receptor^53^, we anticipate that our approach will open opportunities for future optimization of inducible split Cas9 with no toxicity and high activity.

Since several existing Cas9 analogues have been shown to be orthogonal to one another^25^,^32^, using ligand-binding domains from different nuclear receptors could also in principle facilitate simultaneous activation of Cas9 variants to target different gene loci. Furthermore, this method can be combined with combinatorial splitting of Cas9 to enable multiplexed genomic control of the same subset of endogenous genes through temporal control of each split variant. The ability to artificially induce epigenetic marks has already enabled researchers to probe for temporal changes in chromatin state at specific loci, including HP1-mediated heterochromatin spreading^54^. Given the growing list of dCas9-based applications in transcriptional regulation and epigenetic modifications^18^, we anticipate that our approach will similarly allow simultaneous orthogonal induction of different CRISPR systems to provide dynamic control of distinct loci in studies of epigenomic editing.

## Methods

### Plasmid constructs

All plasmid constructs were cloned using Gibson assembly (Supplementary Fig. 8). The constructs of split candidates **A**–**C** encoding the N- and C-terminal fragments of *S. pyogenes* Cas9 were derived from Addgene plasmid 42230 (pX330)^5^. FRB- and FKBP-encoded cDNAs were PCR amplified from FKBP-C-TEV and FRB-N-TEV^55^, and assembled with cDNA encoding split fragment genes into pX330 vector that was digested with *Eco*RI and *Age*I to remove the *S. pyogenes* Cas9 sequence.

To create inducible split CRISPRa constructs, the genes encoding split dCas9 fragments from *S. pyogenes* or *S. areus* were derived from Addgene plasmids 46912 and 61591 (pX601), and VP64- and VPR-encoded cDNAs were PCR amplified from Addgene plasmids 46912 and 63798, respectively. The split CRISPRa constructs were generated by inserting the PCR-amplified fragments into the digested backbone vector from pX330 or pX601 (ref. 33). The cDNA encoding ERT domain was PCR-amplified from pROSA26-CreERT2 plasmid^31^ and cloned by Gibson assembly into split Cas9 plasmids that were digested with either *Eco*RI or *Age*I.

To generate an inducible intein CRISPRa construct (intein-VPR), the cDNA encoding the inducible intein domain and dCas9-VPR were amplified from Addgene plasmids 64192 and 63798, respectively, and cloned by Gibson assembly into the digested backbone vector from pX330. The plasmid pX856-VPR was created by cloning the PCR-amplified VPR-encoded cDNA by Gibson assembly into the Addgene plasmid 62888 (pX856)^24^ that was digested with *Eco*RI.

The fluorescence NHEJ reporter was derived from the previously published fluorescence reporter plasmid encoding *mCherry-TAG-eGFP-HA* (Supplementary Fig. 8)^56^. The cDNA encoding the fluorescence reporter was PCR-amplified and includes a two-nucleotide frameshift mutation following the stop codon. The PCR-amplified fragments were cloned using Gibson Assembly into a pCDNA3.1 vector that was digested with *Bam*HI and *Eco*RI. The plasmids encoding sgRNAs targeting the fluorescence reporter, *RBM20*, *PHOX2B*, *POU5F1* and *GAL4* were constructed by ligating annealed oligonucleotides into *Bbs*I or *Bsa*I site of pX330-derived plasmids. The sequences of oligonucleotides used for sgRNA cloning was included in Supplementary Table 1.

### Cell culture

Adherent HEK 293T (ATCC#CRL-11268) cells were cultured at 37 °C in DMEM (HyClone) medium supplemented with 10% FBS (HyClone) in a 5% CO_2_ atmosphere before transfection. The reporter cell line HEK 293 containing *Gal4-UASx3-GFP-2A-Fluc* operon was derived by lentiviral infection of a previously published plasmid pGL1-Gal4-UAS-dscGFP^9^. The infected cells were FACS sorted and cultured at 37 °C in DMEM (HyClone) containing 10% FBS and puromycin under 5% CO_2_ to maintain the stable polyclonal cell line.

### Fluorescence-based NHEJ assay

HEK 293T cells were plated at ∼0.5 × 10^6^ cells per well in six-well plates and cultured overnight at 37 °C under 5% CO_2_ before transfection. The cells were transiently transfected with TransIT-293 (Mirus Bio) according to the manufacturer's protocol. The fluorescence reporter plasmid encoding mCherry-stop*-eGFP, the second plasmid encoding both N-Cas9 fragment and sgMCE targeting the reporter, and the third plasmid encoding C-Cas9 fragment were transfected at 1:1:1 ratio. For chemically induced assembly of split Cas9, the growth medium was replaced with fresh DMEM medium with 10% FBS and 10 nM rapamycin (Cell Signaling Technology), or DMSO for control, 24 h after transfection. The cells were further incubated at 37 °C in 5% CO_2_ for 48 h before flow analysis. For flow analysis, cells were trypsinized, washed with PBS and analysed on a BD FACSCalibur flow cytometer with a 488-nm argon-ion laser and a 635-nm red-diode laser. Live cell population was gated using SSC/FSC and included only transfected cells, which are mCherry-positive. The proportion of eGFP-positive population compared with total transfected cells was measured by counting cells expressing both mCherry and eGFP. The activity of each split candidate was determined by normalizing the proportion of eGFP-positive population to those in cells expressing wild-type Cas9 and sgMCE and background-subtracted from those in cells expressing sgGAL as negative control.

### ddPCR-based HDR assay

For transfection, 4 × 10^4^ cells were plated into each well of a 96-well plate. One day later, the cells were transfected with DNA, using 0.3 μl of Lipofectamine 2000 (Life Technologies) per well, according to the manufacturer's instructions. For transfections of single Cas9, 90 ng of a plasmid for nucleases and 10 ng of oligonucleotide donor DNA were transfected per well. For split Cas9 nucleases, 45 ng of each vector encoding the N- and C-terminal fragment, respectively, and 10 ng of oligonucleotide donor DNA were transfected per well. One day after transfection, 10 nM rapamycin and/or 10 μM 4OHT were added to the media, and genomic DNA was extracted from the cells 3 days after transfection and resuspended in 30 μl of water per well as described^39^. The ddPCR assays using QX100 Droplet Digital PCR System (Bio-Rad) were conducted with the gRNA, primers and probes for *RBM20* as described^39^. After the PCR amplification step, the droplets were analysed to determine the homologous recombination frequency from the ratio of detected mutant allele droplets to wild-type allele droplets.

### Surveyor assay for quantification of indel mutation rates

To measure the indel mutation rates, HEK 293T cells were plated at ∼0.5 × 10^6^ cells per well in six-well plates and cultured overnight at 37 °C under 5% CO_2_ before transfection. The cells were co-transfected with C-Cas9 encoding plasmid and another plasmid encoding N-Cas9 and sgRNA at 1:1 ratio. For split Cas9 induction, the growth medium was replaced with fresh DMEM medium with 10% FBS, 4OHT (10 μM) and/or rapamycin (10 nM) 24 h after transfection. Cells were then further incubated at 37 °C in 5% CO_2_ for 48 h after induction. Genomic DNA was subsequently extracted using Quick-gDNA Miniprep kit (Zymo) according to the manufacturer's protocol. The genomic target was PCR-amplified using Q5 High-Fidelity DNA polymerase (NEB) for *PHOX2B* (PCR condition: 98 °C, 5 min; (98 °C, 10 s; 65 °C, 30 s; 72 °C, 15 s) × 35 cycles; 72 °C, 10 min). The primers for *PHOX2B* are PHOX2B-fwd (5′-CTCCAGCCACCTTCTCCATA-3′) and PHOX2B-rev (5′-CGCTGAGAAAGCTGAAGGTC-3′). The Surveyor assay was then performed using an adapted protocol^57^. The PCR products were purified using QIAquick PCR purification kit (Qiagen) following the manufacturer's protocol. To produce heteroduplexed DNA, ∼0.5 μg of purified PCR products were diluted with Q5 reaction buffer (NEB) to a final volume of 20 μl, and reannealed using a thermocycler (95 °C, 10 min; 95–85 °C, −2 °C s^−1^; 85 °C, 1 min; 85–75 °C, −0.3 °C/s; 75 °C, 1 min; 75–65 °C, −0.3 °C/s; 65 °C, 1 min; 65–55 °C, −0.3 °C s^−1^; 55 °C, 1 min; 55–45 °C, −0.3 °C s^−1^; 45 °C, 1 min; 45–35 °C, −0.3 °C s^−1^; 35 °C, 1 min; 35–25 °C, −0.3 °C s^−1^; 25 °C, 1 min; 4 °C, hold). The re-annealed heteroduplexed DNA was subsequently digested with Surveyor nuclease S using the Surveyor Mutation Detection Kit (IDT) following the manufacturer's protocol. The digested DNA was analysed by polyacrylamide gel electrophoresis. The gel was stained with SYBR Gold (Life Technologies) and visualized using a Bio-Rad Gel Doc EZ Imager system. Relative band intensities were quantified using the Bio-Rad Image Lab software to determine the percentage of indel mutation induced by split Cas9 following the equation:


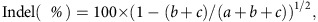


where *a* is the intensity of undigested PCR amplicon, and *b* and *c* are the intensities of the digested products^57^.

### Luciferase-based CRISPRa assay

For CRISPRa-mediated luciferase expression experiments, the stable cell line HEK 293T containing *Gal4-UASx3-GFP-2A-Fluc* operon was plated at ∼0.5 × 10^6^ cells per well in six-well plates and cultured overnight at 37 °C under 5% CO_2_ before transfection. The cells were co-transfected with a plasmid encoding C-dCas9-VP64 or C-dCas9-VPR, and another plasmid encoding N-dCas9 and sgRNA at 1:1 ratio. The transfected cells were trypsinzed and resuspended in fresh DMEM supplemented with 10% FBS 24 h after transfection. Cells were then aliquoted into a 96-well SigmaScreen poly-D-lysine-coated plate and allowed to adhere for 24 h. Rapamycin and/or 4OHT was added to induce CRISPRa activity. Following induction, cells were then further incubated at 37 °C in 5% CO_2_ for 48 h before bioluminescence analysis. For evaluation of CRISPRa activity, cells were lysed with Bright-Glo Luciferase Assay substrate (Promega) according to the manufacturer's instructions and immediately analysed using a SpectraMax M5 multimode plate reader (Molecular Devices). In addition, before cell lysis, GFP fluorescence was imaged using a Zeiss Axio Observer Z1 inverted fluorescence microscope. The luciferase activities mediated by the split constructs were background-substracted from a negative control (cells expressing full-length dCas9-VPR and sgRBM), and normalized against a positive control (cells expressing full-length dCas9-VPR and sgRBM).

### Quantitative RT–PCR

Cells were harvested and total RNA was isolated using a Qiagen RNeasy Mini kit (Qiagen), according to manufacturer's instructions. RNA was converted to cDNA using AMV reverse transcriptase under standard conditions with oligo dT primers and RNasin (Promega). Standard quantitative PCR reactions were prepared according to the manufacturer's instructions (Promega). Reactions were run on a LightCycler thermal cycler (Roche). Gene-specific primer sequences for Actin (5′-GCTACGAGCTGCCTGACG-3′, 5′-GGCTGGAAGAGTGCCTCA-3′) and Oct4 (5′-AGTGAGAGGCAACCTGGAGA-3′, 5′-CGGACCACATCCTTCTCGAG-3′) were used to quantify transcript levels. The fold-increases of mRNA expression levels were normalized against the transcript levels detected in cells expressing full-length dCas9-VPR and sgGAL.

### Data availability

The data that support the findings of this study are available from the corresponding author upon request.

## Additional information

**How to cite this article:** Nguyen, D. P. *et al.* Ligand-binding domains of nuclear receptors facilitate tight control of split CRISPR activity. *Nat. Commun.* 7:12009 doi: 10.1038/ncomms12009 (2016).

## Supplementary Material

Supplementary Information Supplementary Figures 1-8, Supplementary Table 1, Supplementary Methods and Supplementary Reference
