# Supplementary material for: Ligand-binding domains of nuclear receptors facilitate tight control of split CRISPR activity
Source: Nat Commun. 2016 Jul 1;7:12009. doi: 10.1038/ncomms12009 (PMC4932181; doi:10.1038/ncomms12009)
Supplement: Supplementary Information — Supplementary Figures 1-8, Supplementary Table 1, Supplementary Methods and Supplementary Reference [file ncomms12009-s1.pdf]

Supplementary Fig. 1

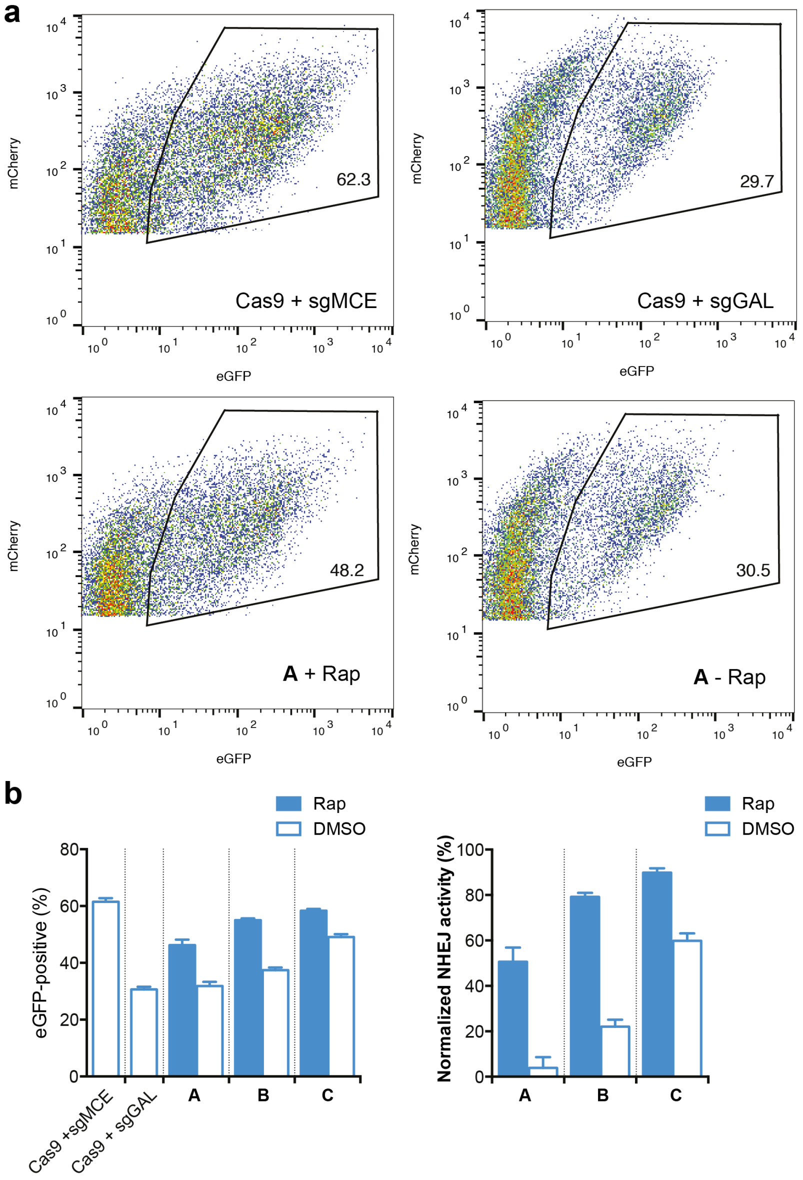

Supplementary Figure 1. Fluorescence-based NHEJ Assay. (**a**) Representative FACS analyses of fluorescence-based reporter. HEK293T cells were gated based on red fluoresecence to include only mCherry-positive cells for analysis. The percentage of cells expressing both mCherry and eGFP is indicated. (Top left) Cells expressing reporter, wild-type Cas9 and reporter-targeting sgRNA (sgMCE). (Top right) Cells expressing reporter, wild-type Cas9 and sgRNA negative control (sgGAL). (Bottom left) Rapamycin-induced cells expressing reporter and split-C. (Bottom right) Non-induced cells expressing reporter and split-C. (**b**) Activities of split candidates in the fluorescence-based NHEJ assay. (Left) Plots of percentages of cells expressing both mCherry and eGFP. (Right) Plots of NHEJ activity which was background-substracted from a negative control (cells expressing reporter, wild-type Cas9 and sgGAL), and normalized against a positive control (cells expressing reporter, wild-type Cas9 and sgMCE).

Supplementary Fig. 2

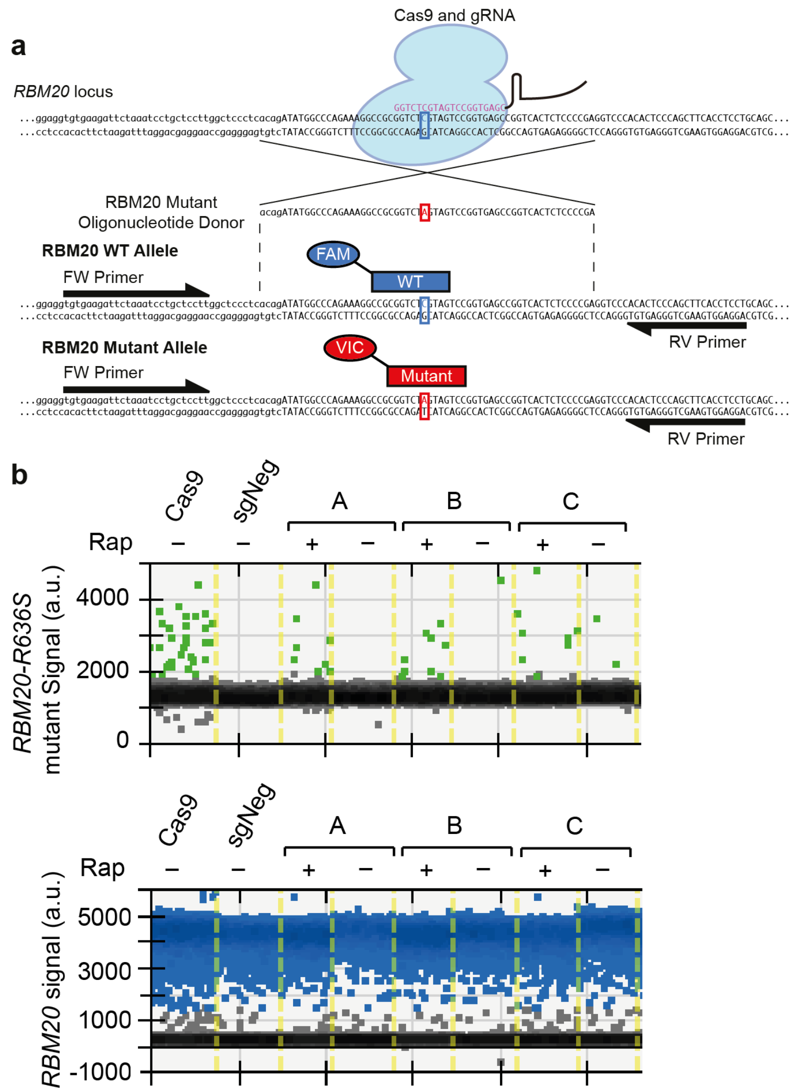

Supplementary Figure 2. (**a**) Mutagenesis and detection strategy for *RBM20*, showing a pair of Cas9 and sgRNA, containing the 20-nucleotide guide sequence as depicted, designed to target the mutation site in *RBM20*. Allele-specific probes conjugated with either FAM or VIC, a 60-nt single-stranded oligonucleotide DNA donor, and locations of a common primer pair are also shown. (**b**) Representative raw droplet data of the ddPCR assay on split candidates. Yellow lines indicate borders between different samples. Colored dots represent droplets containing detected alleles. (Top) Droplets containing mutagenized *RBM20* allele *R636S* (green). (Bottom) Dropets containing wildtype *RBM20* allele (blue). The mutant allele frequency is measured by determining the ratio of mutant-allele droplets to wildtype-allele droplets.

Supplementary Fig. 3

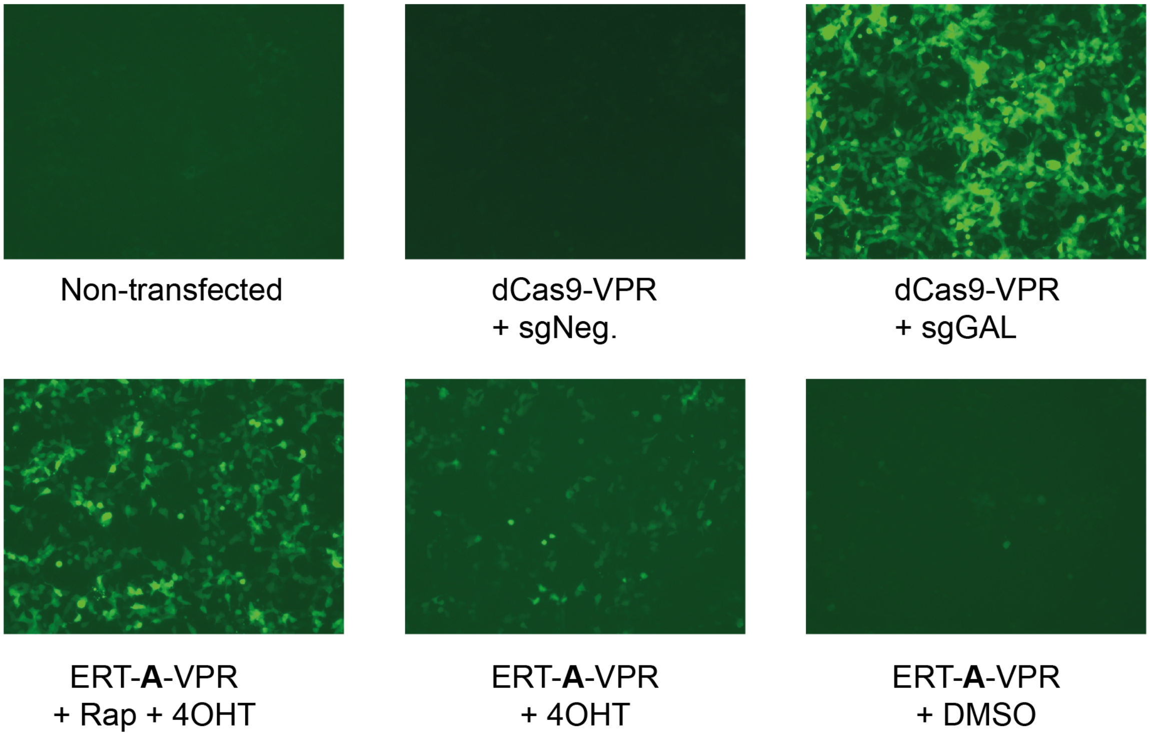

Supplementary Figure 3. Fluorescence microscopy of cells expressing *eGFP-2A-Fluc* reporter and dCas9-VPR or ERT-**A**-VPR with 4OHT (10μM) and/or rapamycin (10 nM). The negative sgRNA control used in the experiment was the RMB20-targeting sgRNA, sgRBM.

Supplementary Fig. 4

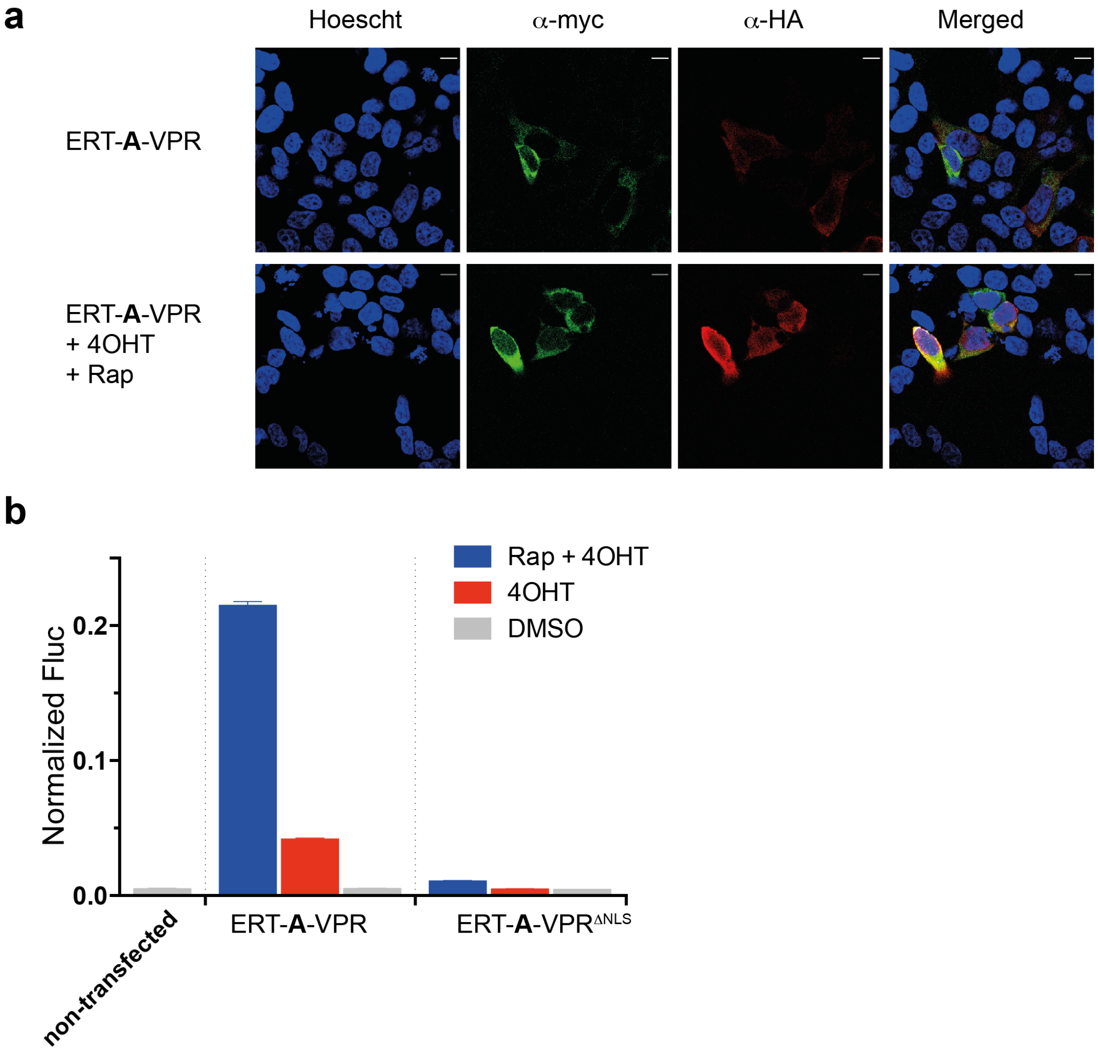

Supplementary Figure 4. Luciferase-based CRISPRa assay of inducible split Cas9 constructs. (**a**) Immunofluorescent staining of HEK293T cells expressing ERT-**A**-VPR induced with 4-OHT (2 μM) and Rap (10 nM). The fixed cells were stained with nuclear-staining Hoescht dye (blue), α-myc (green) and α-HA (red) antibodies. Top: non-induced; bottom: induced cells. Scale bar: 10 μm. (**b**) NLS sequences are required for efficient transcriptional activation by ERT-**A**-VPR. The NLS sequences were removed from the VPR domain of ERT-**A**-VPR to create the ERT-**A**-VPR$^{\Delta NLS}$ construct. HEK293T Cells expressing either ERT-**A**-VPR or ERT-**A**-VPR$^{\Delta NLS}$ were induced by the addition of 4OHT (10 μM) and rapamycin (10 nM). The luciferase activities are normalized against the activity exhibited from cells expressing dCas9-VPR. The data are presented as mean mean ± standard deviation for 3 biological replicates.

Supplementary Fig. 5

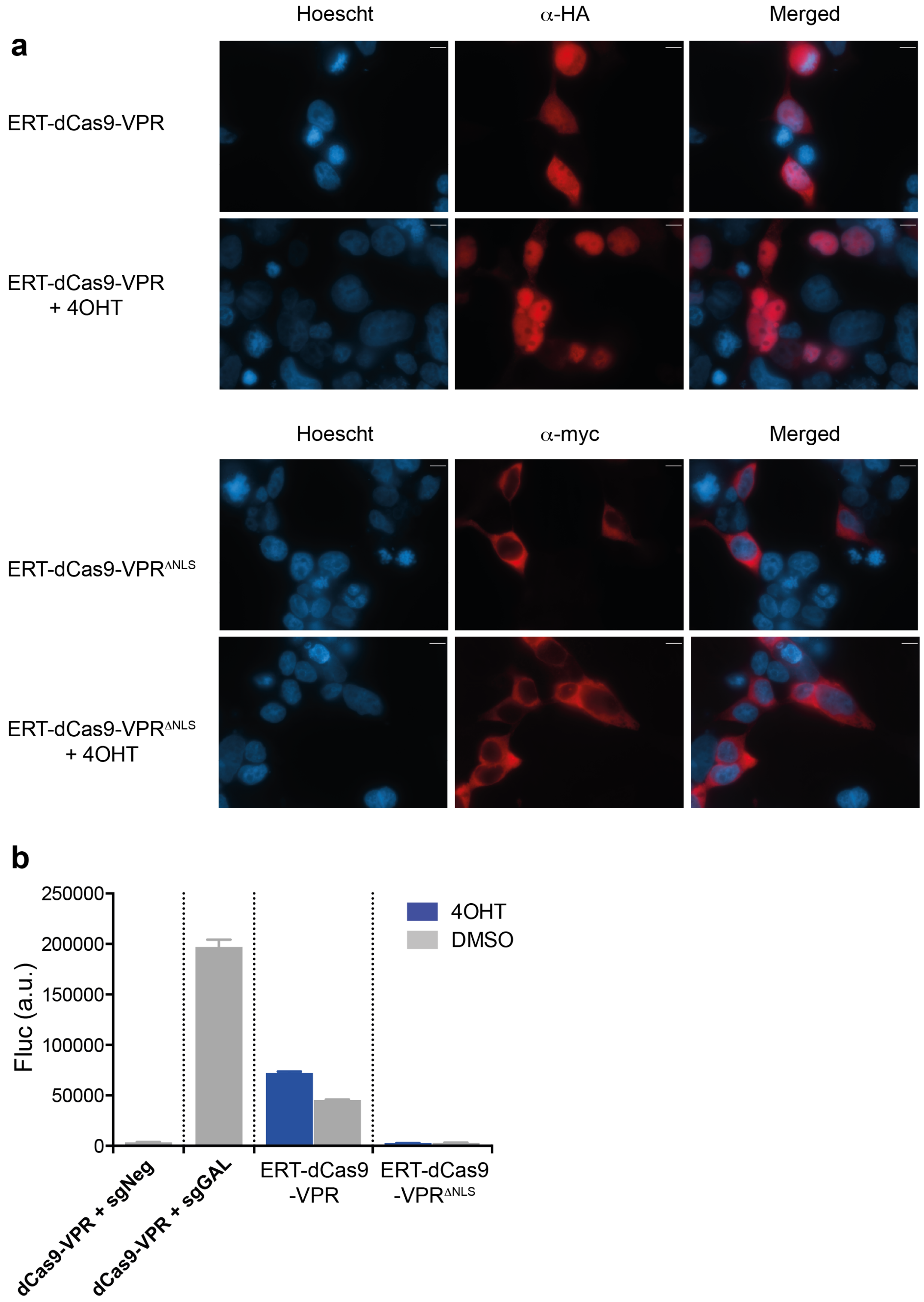

Supplementary Figure 5. Luciferase-based CRISPRa assay of ERT-fused full-length dCas9 constructs. (**a**) Immunofluorescent staining of HEK293T cells expressing ERT-dCas9-VPR or ERT-dCas9-VPR$^{\Delta NLS}$ induced with 4-OHT (10 μM). The ERT domain was fused to the N-termini of dCas9-VPR and dCas9-VPR$^{\Delta NLS}$ to create ERT-dCas9-VPR and ERT-**A**-VPR$^{\Delta NLS}$ respectively. The fixed cells were stained with nuclear-staining Hoescht dye (blue) and α-HA or α-myc (red) antibody. Top: non-induced; bottom: induced cells. Scale bar: 10 μm. (**b**) Comparison of dCas9-VPR, ERT-dCas9-VPR and ERT-dCas9-VPR$^{\Delta NLS}$. 4OHT (10 μM) was used to induce ERT-dCas9-VPR activity. The luciferase activities are presented as mean ± standard deviation for 3 biological replicates.

Supplementary Fig. 6

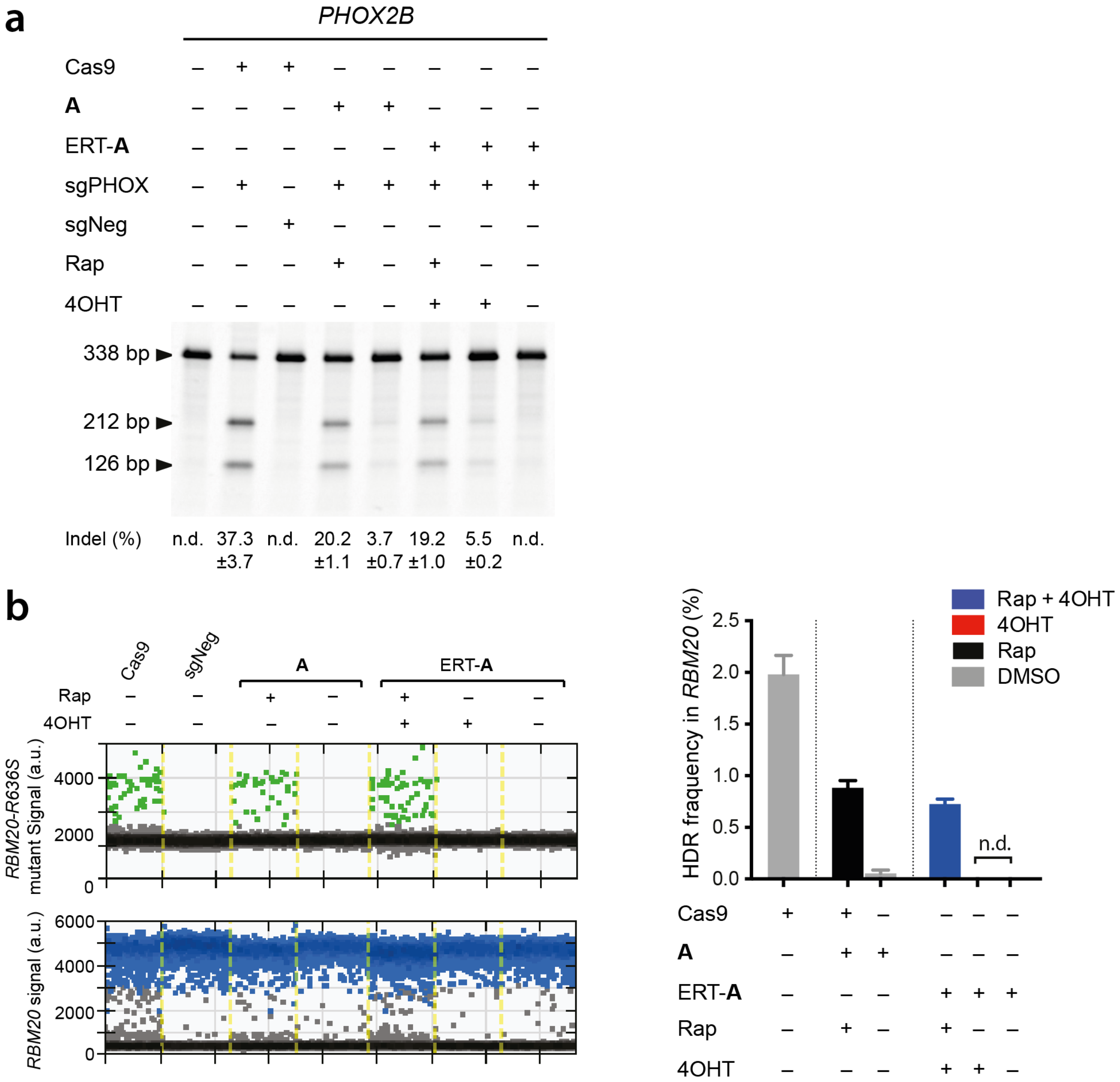

Supplementary Figure 6. ERT-A facilitates tight control of endonuclease activity in split Cas9. (**a**) Surveyor assay of indels mediated by ERT-**A** at *PHOX2B* gene locus. Indel rates are presented as means ± standard deviation for 3 biological replicates. N.d., not detected. (**b**) HDR activity of ERT-**A** at the *RBM20* gene locus (n = 3 biological replicates). N.d., not detected. Left panels show the representative raw droplet data of the ddPCR assay on cells expressing split-**A** or ERT-**A**. Top left: Droplets containing the *RBM20* allele *R636S* (green); Bottom left: Droplets containing the wildtype *RBM20* allele (blue). The data of HDR efficiency (right) are displayed as mean ± standard deviation for 3 biological replicates.

Supplementary Fig. 7

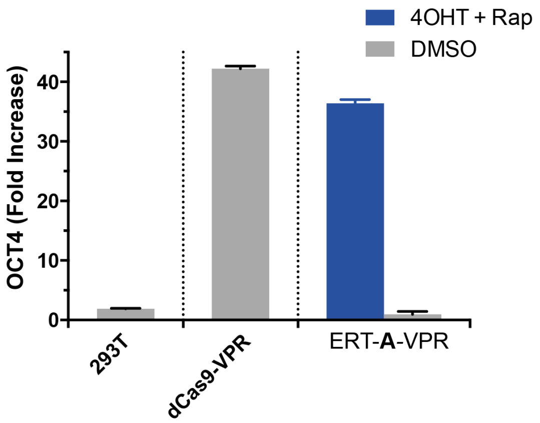

Supplementary Figure 7. Transcriptional activation of endogenous Oct4 expression in HEK293T cells by ERT-**A**-VPR. Cells expressing ERT-**A**-VPR were induced with 4OHT (10 μM) and Rap (10 nM). The mRNA expression levels relative to a negative control (cells expressing dCas9-VPR and sgRBM) were presented as mean ± standard deviation for 3 biological replicates.

Supplementary Figure 8. Nucleotide and amino acid sequences of constructs used in this study.

(**A**) Amino acid sequences of split Cas9 candidates:

(**i**) Split-**A**:

N-Cas9 fragment:

Blue = FLAG tag; Orange = NLS; Green = Gly-Ser linker; Dark red = FRB

```
MDYKDHDGDYKDHDIDYKDDDDKMAPKKKRKVGIHGVPAADKKYSIGLDIGTNSVGWAVI
TDEYKVPSKKFKVLGNTDRHSIKKNLIGALLFDSGETAEATRLKRTARRRYTRRKNRICY
LQEIFSNEMAKVDDSFFHRLEESFLVEEDKKHERHPIFGNIVDEVAYHEKYPTIYHLRKK
LVDSTDKADLRLIYLALAHMIKFRGHFLIEGDLGGGGSGGGGSEMWHEGLEEASRLYFGE
RNVKGMFEVLEPLHAMMERGPQTLKETSFNQAYGRDLMEAQEWCRKYMKSGNVKDLTQAW
DLYYHVFRRISKQ
```

C-Cas9 fragment:

Blue = myc tag; Orange = NLS; Green = Gly-Ser linker; Dark red = FKBP

```
MEQKLISEEDLLEGVQVETISPGDGRTFPKRGQTCVVHYTGMLEDGKKFDSSRDRNKPFK
FMLGKQEVIRGWEEGVAQMSVGQRAKLTISPDYAYGATGHPGIIPPHATLVFDVELLKLE
GGGGSGGGGSVNTEITKAPLSASMIKRYDEHHQDLTLLKALVRQQLPEKYKEIFFDQSKN
GYAGYIDGGASQEEFYKFIKPILEKMDGTEELLVKLNREDLLRKQRTFDNGSIPHQIHLG
ELHAILRRQEDFYPFLKDNREKIEKILTFRIPYYVGPLARGNSRFAWMTRKSEETITPWN
FEEVVDKGASAQSFIERMTNFDKNLPNEKVLPKHSLLYEYFTVYNELTKVKYVTEGMRKP
AFLSGEQKKAIVDLLFKTNRKVTVKQLKEDYFKKIECFDSVEISGVEDRFNASLGTYHDL
LKIIKDKDFLDNEENEDILEDIVLTLTLFEDREMIEERLKTYAHLFDDKVMKQLKRRRYT
GWGRLSRKLINGIRDKQSGKTILDFLKSDGFANRNFMQLIHDDSLTFKEDIQKAQVSGQG
DSLHEHIANLAGSPAIKKGILQTVKVVDELVKVMGRHKPENIVIEMARENQTTQKGQKNS
RERMKRIEEGIKELGSQILKEHPVENTQLQNEKLYLYYLQNGRDMYVDQELDINRLSDYD
VDHIVPQSFLKDDSIDNKVLTRSDKNRGKSDNVPSEEVVKKMKNYWRQLLNAKLITQRKF
DNLTKAERGGLSELDKAGFIKRQLVETRQITKHVAQILDSRMNTKYDENDKLIREVKVIT
LKSKLVSDFRKDFQFYKVREINNYHHAHDAYLNAVVGTALIKKYPKLESEFVYGDYKVYD
VRKMIAKSEQEIGKATAKYFFYSNIMNFFKTEITLANGEIRKRPLIETNGETGEIVWDKG
RDFATVRKVLSMPQVNIVKKTEVQTGGFSKESILPKRNSDKLIARKKDWDPKKYGGFDSP
TVAYSVLVVAKVEKGKSKKLKSVKELLGITIMERSSFEKNPIDFLEAKGYKEVKKDLIIK
LPKYSLFELENGRKRMLASAGELQKGNELALPSKYVNFLYLASHYEKLKGSPEDNEQKQL
FVEQHKHYLDEIIEQISEFSKRVILADANLDKVLSAYNKHRDKPIREQAENIIHLFTLTN
LGAPAAFKYFDTTIDRKRYTSTKEVLDATLIHQSITGLYETRIDLSQLGGDKRPAATKKA
GQAKKKK
```

(**ii**) Split-**B**:

N-Cas9 fragment:

Blue = FLAG tag; Orange = NLS; Green = Gly-Ser linker; Dark red = FRB

```
MDYKDHDGDYKDHDIDYKDDDDKMAPKKKRKVGIHGVPAADKKYSIGLDIGTNSVGWAVI
TDEYKVPSKKFKVLGNTDRHSIKKNLIGALLFDSGETAEATRLKRTARRRYTRRKNRICY
LQEIFSNEMAKVDDSFFHRLEESFLVEEDKKHERHPIFGNIVDEVAYHEKYPTIYHLRKK
LVDSTDKADLRLIYLALAHMIKFRGHFLIEGDLNPDNGGGGSGGGGSEMWHEGLEEASRL
YFGERNVKGMFEVLEPLHAMMERGPQTLKETSFNQAYGRDLMEAQEWCRKYMKSGNVKDL
TQAWDLYYHVFRRISKQ
```

C-Cas9 fragment:

Blue = myc tag; Orange = NLS; Green = Gly-Ser linker; Dark red = FKBP

```
MEQKLISEEDLLEGVQVETISPGDGRTFPKRGQTCVVHYTGMLEDGKKFDSSRDRNKPFK
FMLGKQEVIRGWEEGVAQMSVGQRAKLTISPDYAYGATGHPGIIPPHATLVFDVELLKLE
```

```
GGGGSGGGGSSDVDKLFIQLVQTYNQLFEENPINASGVDAKAILSARLSKSRRLENLIAQ
LPGEKKNGLFGNLIALSLGLTPNFKSNFDLAEDAKLQLSKDTYDDDLDNLLAQIGDQYAD
LFLAAKNLSDAILLSDILRVNTEITKAPLSASMIKRYDEHHQDLTLLKALVRQQLPEKYK
EIFFDQSKNGYAGYIDGGASQEEFYKFIKPILEKMDGTEELLVKLNREDLLRKQRTFDNG
SIPHQIHLGELHAILRRQEDFYPFLKDNREKIEKILTFRIPYYVGPLARGNSRFAWMTRK
SEETITPWNFEEVVDKGASAQSFIERMTNFDKNLPNEKVLPKHSLLYEYFTVYNELTKVK
YVTEGMRKPAFLSGEQKKAIVDLLFKTNRKVTVKQLKEDYFKKIECFDSVEISGVEDRFN
ASLGTYHDLLKIIKDKDFLDNEENEDILEDIVLTLTLFEDREMIEERLKTYAHLFDDKVM
KQLKRRRYTGWGRLSRKLINGIRDKQSGKTILDFLKSDGFANRNFMQLIHDDSLTFKEDI
QKAQVSGQGDSLHEHIANLAGSPAIKKGILQTVKVVDELVKVMGRHKPENIVIEMARENQ
TTQKGQKNSRERMKRIEEGIKELGSQILKEHPVENTQLQNEKLYLYYLQNGRDMYVDQEL
DINRLSDYDVDHIVPQSFLKDDSIDNKVLTRSDKNRGKSDNVPSEEVVKKMKNYWRQLLN
AKLITQRKFDNLTKAERGGLSELDKAGFIKRQLVETRQITKHVAQILDSRMNTKYDENDK
LIREVKVITLKSKLVSDFRKDFQFYKVREINNYHHAHDAYLNAVVGTALIKKYPKLESEF
VYGDYKVYDVRKMIAKSEQEIGKATAKYFFYSNIMNFFKTEITLANGEIRKRPLIETNGE
TGEIVWDKGRDFATVRKVLSMPQVNIVKKTEVQTGGFSKESILPKRNSDKLIARKKDWDP
KKYGGFDSPTVAYSVLVVAKVEKGKSKKLKSVKELLGITIMERSSFEKNPIDFLEAKGYK
EVKKDLIIKLPKYSLFELENGRKRMLASAGELQKGNELALPSKYVNFLYLASHYEKLKGS
PEDNEQKQLFVEQHKHYLDEIIEQISEFSKRVILADANLDKVLSAYNKHRDKPIREQAEN
IIHLFTLTNLGAPAAFKYFDTTIDRKRYTSTKEVLDATLIHQSITGLYETRIDLSQLGGD
KRPAATKKAGQAKKKK
```

(iii) Split-**C**:

N-Cas9 fragment:

Blue = FLAG tag; Orange = NLS; Green = Gly-Ser linker; Dark red = FRB

```
MDYKDHDGDYKDHDIDYKDDDDKMAPKKKRKVGIHGVPAADKKYSIGLDIGTNSVGWAVI
TDEYKVPSKKFKVLGNTDRHSIKKNLIGALLFDSGETAEATRLKRTARRRYTRRKNRICY
LQEIFSNEMAKVDDSFFHRLEESFLVEEDKKHERHPIFGNIVDEVAYHEKYPTIYHLRKK
LVDSTDKADLRLIYLALAHMIKFRGHFLIEGDLNPDNSDVDKLFIQLVQTYNQLFEENPI
NASGGGGSGGGGSEMWHEGLEEASRLYFGERNVKGMFEVLEPLHAMMERGPQTLKETSFN
QAYGRDLMEAQEWCRKYMKSGNVKDLTQAWDLYYHVFRRISKQ
```

C-Cas9 fragment:

Blue = myc tag; Orange = NLS; Green = Gly-Ser linker; Dark red = FKBP

```
MEQKLISEEDLLEGVQVETISPGDGRTFPKRGQTCVVHYTGMLEDGKKFDSSRDRNKPFK
FMLGKQEVIRGWEEGVAQMSVGQRAKLTISPDYAYGATGHPGIIPPHATLVFDVELLKLE
GGGGSGGGGSGVDAKAILSARLSKSRRLENLIAQLPGEKKNGLFGNLIALSLGLTPNFKS
NFDLAEDAKLQLSKDTYDDDLDNLLAQIGDQYADLFLAAKNLSDAILLSDILRVNTEITK
APLSASMIKRYDEHHQDLTLLKALVRQQLPEKYKEIFFDQSKNGYAGYIDGGASQEEFYK
FIKPILEKMDGTEELLVKLNREDLLRKQRTFDNGSIPHQIHLGELHAILRRQEDFYPFLK
DNREKIEKILTFRIPYYVGPLARGNSRFAWMTRKSEETITPWNFEEVVDKGASAQSFIER
MTNFDKNLPNEKVLPKHSLLYEYFTVYNELTKVKYVTEGMRKPAFLSGEQKKAIVDLLFK
TNRKVTVKQLKEDYFKKIECFDSVEISGVEDRFNASLGTYHDLLKIIKDKDFLDNEENED
ILEDIVLTLTLFEDREMIEERLKTYAHLFDDKVMKQLKRRRYTGWGRLSRKLINGIRDKQ
SGKTILDFLKSDGFANRNFMQLIHDDSLTFKEDIQKAQVSGQGDSLHEHIANLAGSPAIK
KGILQTVKVVDELVKVMGRHKPENIVIEMARENQTTQKGQKNSRERMKRIEEGIKELGSQ
ILKEHPVENTQLQNEKLYLYYLQNGRDMYVDQELDINRLSDYDVDHIVPQSFLKDDSIDN
KVLTRSDKNRGKSDNVPSEEVVKKMKNYWRQLLNAKLITQRKFDNLTKAERGGLSELDKA
GFIKRQLVETRQITKHVAQILDSRMNTKYDENDKLIREVKVITLKSKLVSDFRKDFQFYK
VREINNYHHAHDAYLNAVVGTALIKKYPKLESEFVYGDYKVYDVRKMIAKSEQEIGKATA
KYFFYSNIMNFFKTEITLANGEIRKRPLIETNGETGEIVWDKGRDFATVRKVLSMPQVNI
VKKTEVQTGGFSKESILPKRNSDKLIARKKDWDPKKYGGFDSPTVAYSVLVVAKVEKGKS
KKLKSVKELLGITIMERSSFEKNPIDFLEAKGYKEVKKDLIIKLPKYSLFELENGRKRML
ASAGELQKGNELALPSKYVNFLYLASHYEKLKGSPEDNEQKQLFVEQHKHYLDEIIEQIS
EFSKRVILADANLDKVLSAYNKHRDKPIREQAENIIHLFTLTNLGAPAAFKYFDTTIDRK
RYTSTKEVLDATLIHQSITGLYETRIDLSQLGGDKRPAATKKAGQAKKKK
```

(**iv**) Split ERT-**A**:

N-Cas9 fragment:

Blue = FLAG tag; Orange = NLS; Green = Gly-Ser linker; Dark red = FRB; Yellow = ERT; Turquoise = HA tag

```
MDYKDHDGDYKDHDIDYKDDDDKMAPKKKRKVGIHGVPAADKKYSIGLDIGTNSVGWAVI
TDEYKVPSKKFKVLGNTDRHSIKKNLIGALLFDSGETAEATRLKRTARRRYTRRKNRICY
LQEIFSNEMAKVDDSFFHRLEESFLVEEDKKHERHPIFGNIVDEVAYHEKYPTIYHLRKK
LVDSTDKADLRLIYLALAHMIKFRGHFLIEGDLGGGGSGGGGSEMWHEGLEEASRLYFGE
RNVKGMFEVLEPLHAMMERGPQTLKETSFNQAYGRDLMEAQEWCRKYMKSGNVKDLTQAW
DLYYHVFRRISKQGGSLEPSAGDMRAANLWPSPLMIKRSKKNSLALSLTADQMVSALLDA
EPPILYSEYDPTRPFSEASMMGLLTNLADRELVHMINWAKRVPGFVDLTLHDQVHLLECA
WLEILMIGLVWRSMEHPVKLLFAPNLLLDRNQGKCVEGMVEIFDMLLATSSRFRMMNLQG
EEFVCLKSIILLNSGVYTFLSSTLKSLEEKDHIHRVLDKITDTLIHLMAKAGLTLQQQHQ
RLAQLLLILSHIRHMSNKGMEHLYSMKCKNVVPLYDLLLEAADAHRLHAPTSRGGASVEE
TDQSHLATAGSTSSHSLQKYYITGEAEGFPATAGGSYPYDVPDYA
```

C-Cas9 fragment:

Blue = myc tag; Orange = NLS; Green = Gly-Ser linker; Dark red = FKBP; Yellow = ERT

```
MEQKLISEEDLLESAGDMRAANLWPSPLMIKRSKKNSLALSLTADQMVSALLDAEPPILY
SEYDPTRPFSEASMMGLLTNLADRELVHMINWAKRVPGFVDLTLHDQVHLLECAWLEILM
IGLVWRSMEHPVKLLFAPNLLLDRNQGKCVEGMVEIFDMLLATSSRFRMMNLQGEEFVCL
KSIILLNSGVYTFLSSTLKSLEEKDHIHRVLDKITDTLIHLMAKAGLTLQQQHQRLAQLL
LILSHIRHMSNKGMEHLYSMKCKNVVPLYDLLLEAADAHRLHAPTSRGGASVEETDQSHL
ATAGSTSSHSLQKYYITGEAEGFPATAGGGGSGGGGSGVQVETISPGDGRTFPKRGQTCV
VHYTGMLEDGKKFDSSRDRNKPFKFMLGKQEVIRGWEEGVAQMSVGQRAKLTISPDYAYG
ATGHPGIIPPHATLVFDVELLKLEGGGGSGGGGSNPDNSDVDKLFIQLVQTYNQLFEENP
INASGVDAKAILSARLSKSRRLENLIAQLPGEKKNGLFGNLIALSLGLTPNFKSNFDLAE
DAKLQLSKDTYDDDLDNLLAQIGDQYADLFLAAKNLSDAILLSDILRVNTEITKAPLSAS
MIKRYDEHHQDLTLLKALVRQQLPEKYKEIFFDQSKNGYAGYIDGGASQEEFYKFIKPIL
EKMDGTEELLVKLNREDLLRKQRTFDNGSIPHQIHLGELHAILRRQEDFYPFLKDNREKI
EKILTFRIPYYVGPLARGNSRFAWMTRKSEETITPWNFEEVVDKGASAQSFIERMTNFDK
NLPNEKVLPKHSLLYEYFTVYNELTKVKYVTEGMRKPAFLSGEQKKAIVDLLFKTNRKVT
VKQLKEDYFKKIECFDSVEISGVEDRFNASLGTYHDLLKIIKDKDFLDNEENEDILEDIV
LTLTLFEDREMIEERLKTYAHLFDDKVMKQLKRRRYTGWGRLSRKLINGIRDKQSGKTIL
DFLKSDGFANRNFMQLIHDDSLTFKEDIQKAQVSGQGDSLHEHIANLAGSPAIKKGILQT
VKVVDELVKVMGRHKPENIVIEMARENQTTQKGQKNSRERMKRIEEGIKELGSQILKEHP
VENTQLQNEKLYLYYLQNGRDMYVDQELDINRLSDYDVDHIVPQSFLKDDSIDNKVLTRS
DKNRGKSDNVPSEEVVKKMKNYWRQLLNAKLITQRKFDNLTKAERGGLSELDKAGFIKRQ
LVETRQITKHVAQILDSRMNTKYDENDKLIREVKVITLKSKLVSDFRKDFQFYKVREINN
YHHAHDAYLNAVVGTALIKKYPKLESEFVYGDYKVYDVRKMIAKSEQEIGKATAKYFFYS
NIMNFFKTEITLANGEIRKRPLIETNGETGEIVWDKGRDFATVRKVLSMPQVNIVKKTEV
QTGGFSKESILPKRNSDKLIARKKDWDPKKYGGFDSPTVAYSVLVVAKVEKGKSKKLKSV
KELLGITIMERSSFEKNPIDFLEAKGYKEVKKDLIIKLPKYSLFELENGRKRMLASAGEL
QKGNELALPSKYVNFLYLASHYEKLKGSPEDNEQKQLFVEQHKHYLDEIIEQISEFSKRV
ILADANLDKVLSAYNKHRDKPIREQAENIIHLFTLTNLGAPAAFKYFDTTIDRKRYTSTK
EVLDATLIHQSITGLYETRIDLSQLGGDKRPAATKKAGQAKKKK
```

(**v**) Split ERT-**C**:

N-Cas9 fragment:

Blue = FLAG tag; Orange = NLS; Green = Gly-Ser linker; Dark red = FRB; Yellow = ERT; Turquoise = HA tag

```
MDYKDHDGDYKDHDIDYKDDDDKMAPKKKRKVGIHGVPAADKKYSIGLDIGTNSVGWAVI
TDEYKVPSKKFKVLGNTDRHSIKKNLIGALLFDSGETAEATRLKRTARRRYTRRKNRICY
LQEIFSNEMAKVDDSFFHRLEESFLVEEDKKHERHPIFGNIVDEVAYHEKYPTIYHLRKK
LVDSTDKADLRLIYLALAHMIKFRGHFLIEGDLNPDNSDVDKLFIQLVQTYNQLFEENPI
NASGGGGSGGGGSEMWHEGLEEASRLYFGERNVKGMFEVLEPLHAMMERGPQTLKETSFN
QAYGRDLMEAQEWCRKYMKSGNVKDLTQAWDLYYHVFRRISKQGGSLEPSAGDMRAANLW
PSPLMIKRSKKNSLALSLTADQMVSALLDAEPPILYSEYDPTRPFSEASMMGLLTNLADR
ELVHMINWAKRVPGFVDLTLHDQVHLLECAWLEILMIGLVWRSMEHPVKLLFAPNLLLDR
NQGKCVEGMVEIFDMLLATSSRFRMMNLQGEEFVCLKSIILLNSGVYTFLSSTLKSLEEK
DHIHRVLDKITDTLIHLMAKAGLTLQQQHQRLAQLLLILSHIRHMSNKGMEHLYSMKCKN
VVPLYDLLLEAADAHRLHAPTSRGGASVEETDQSHLATAGSTSSHSLQKYYITGEAEGFP
ATAGGSYPYDVPDYA
```

C-Cas9 fragment:

Blue = myc tag; Orange = NLS; Green = Gly-Ser linker; Dark red = FKBP; Yellow = ERT

```
MEQKLISEEDLLESAGDMRAANLWPSPLMIKRSKKNSLALSLTADQMVSALLDAEPPILY
SEYDPTRPFSEASMMGLLTNLADRELVHMINWAKRVPGFVDLTLHDQVHLLECAWLEILM
IGLVWRSMEHPVKLLFAPNLLLDRNQGKCVEGMVEIFDMLLATSSRFRMMNLQGEEFVCL
KSIILLNSGVYTFLSSTLKSLEEKDHIHRVLDKITDTLIHLMAKAGLTLQQQHQRLAQLL
LILSHIRHMSNKGMEHLYSMKCKNVVPLYDLLLEAADAHRLHAPTSRGGASVEETDQSHL
ATAGSTSSHSLQKYYITGEAEGFPATAGGGGSGGGGSGVQVETISPGDGRTFPKRGQTCV
VHYTGMLEDGKKFDSSRDRNKPFKFMLGKQEVIRGWEEGVAQMSVGQRAKLTISPDYAYG
ATGHPGIIPPHATLVFDVELLKLEGGGGSGGGGSGVDAKAILSARLSKSRRLENLIAQLP
GEKKNGLFGNLIALSLGLTPNFKSNFDLAEDAKLQLSKDTYDDDLDNLLAQIGDQYADLF
LAAKNLSDAILLSDILRVNTEITKAPLSASMIKRYDEHHQDLTLLKALVRQQLPEKYKEI
FFDQSKNGYAGYIDGGASQEEFYKFIKPILEKMDGTEELLVKLNREDLLRKQRTFDNGSI
PHQIHLGELHAILRRQEDFYPFLKDNREKIEKILTFRIPYYVGPLARGNSRFAWMTRKSE
ETITPWNFEEVVDKGASAQSFIERMTNFDKNLPNEKVLPKHSLLYEYFTVYNELTKVKYV
TEGMRKPAFLSGEQKKAIVDLLFKTNRKVTVKQLKEDYFKKIECFDSVEISGVEDRFNAS
LGTYHDLLKIIKDKDFLDNEENEDILEDIVLTLTLFEDREMIEERLKTYAHLFDDKVMKQ
LKRRRYTGWGRLSRKLINGIRDKQSGKTILDFLKSDGFANRNFMQLIHDDSLTFKEDIQK
AQVSGQGDSLHEHIANLAGSPAIKKGILQTVKVVDELVKVMGRHKPENIVIEMARENQTT
QKGQKNSRERMKRIEEGIKELGSQILKEHPVENTQLQNEKLYLYYLQNGRDMYVDQELDI
NRLSDYDVDHIVPQSFLKDDSIDNKVLTRSDKNRGKSDNVPSEEVVKKMKNYWRQLLNAK
LITQRKFDNLTKAERGGLSELDKAGFIKRQLVETRQITKHVAQILDSRMNTKYDENDKLI
REVKVITLKSKLVSDFRKDFQFYKVREINNYHHAHDAYLNAVVGTALIKKYPKLESEFVY
GDYKVYDVRKMIAKSEQEIGKATAKYFFYSNIMNFFKTEITLANGEIRKRPLIETNGETG
EIVWDKGRDFATVRKVLSMPQVNIVKKTEVQTGGFSKESILPKRNSDKLIARKKDWDPKK
YGGFDSPTVAYSVLVVAKVEKGKSKKLKSVKELLGITIMERSSFEKNPIDFLEAKGYKEV
KKDLIIKLPKYSLFELENGRKRMLASAGELQKGNELALPSKYVNFLYLASHYEKLKGSPE
DNEQKQLFVEQHKHYLDEIIEQISEFSKRVILADANLDKVLSAYNKHRDKPIREQAENII
HLFTLTNLGAPAAFKYFDTTIDRKRYTSTKEVLDATLIHQSITGLYETRIDLSQLGGDKR
PAATKKAGQAKKKK
```

(**B**) Amino acid sequences of split dCas9:

(**i**) Split **A**(-VP64):

N-dCas9 fragment:

Blue = HA tag; Orange = NLS; Green = Gly-Ser linker; Dark red = FRB

```
MYPYDVPDYASPKKKRKVEASDKKYSIGLDIGTNSVGWAVITDEYKVPSKKFKVLGNTDR
HSIKKNLIGALLFDSGETAEATRLKRTARRRYTRRKNRICYLQEIFSNEMAKVDDSFFHR
LEESFLVEEDKKHERHPIFGNIVDEVAYHEKYPTIYHLRKKLVDSTDKADLRLIYLALAH
MIKFRGHFLIEGDLGGGGSGGGGSEMWHEGLEEASRLYFGERNVKGMFEVLEPLHAMMER
GPQTLKETSFNQAYGRDLMEAQEWCRKYMKSGNVKDLTQAWDLYYHVFRRISKQ
```

C-dCas9 fragment:

Blue = myc tag; Orange = NLS; Green = Gly-Ser linker; Dark red = FKBP; Turquoise = VP64; Blue = BFP

MEQKLISEEDLLEGVQVETISPGDGRTFPKRGQTCVVHYTGMLEDGKKFDSSRDRNKPFK
FMLGKQEVIRGWEEGVAQMSVGQRAKLTISPDYAYGATGHPGIIPPHATLVFDVELLKLE
GGGGSGGGGSNPDNSDVDKLFIQLVQTYNQLFEENPINASGVDAKAILSARLSKSRRLEN
LIAQLPGEKKNGLFGNLIALSLGLTPNFKSNFDLAEDAKLQLSKDTYDDDLDNLLAQIGD
QYADLFLAAKNLSDAILLSDILRVNTEITKAPLSASMIKRYDEHHQDLTLLKALVRQQLP
EKYKEIFFDQSKNGYAGYIDGGASQEEFYKFIKPILEKMDGTEELLVKLNREDLLRKQRT
FDNGSIPHQIHLGELHAILRRQEDFYPFLKDNREKIEKILTFRIPYYVGPLARGNSRFAW
MTRKSEETITPWNFEEVVDKGASAQSFIERMTNFDKNLPNEKVLPKHSLLYEYFTVYNEL
TKVKYVTEGMRKPAFLSGEQKKAIVDLLFKTNRKVTVKQLKEDYFKKIECFDSVEISGVE
DRFNASLGTYHDLLKIIKDKDFLDNEENEDILEDIVLTLTLFEDREMIEERLKTYAHLFD
DKVMKQLKRRRYTGWGRLSRKLINGIRDKQSGKTILDFLKSDGFANRNFMQLIHDDSLTF
KEDIQKAQVSGQGDSLHEHIANLAGSPAIKKGILQTVKVVDELVKVMGRHKPENIVIEMA
RENQTTQKGQKNSRERMKRIEEGIKELGSQILKEHPVENTQLQNEKLYLYYLQNGRDMYV
DQELDINRLSDYDVDAIVPQSFLKDDSIDNKVLTRSDKNRGKSDNVPSEEVVKKMKNYWR
QLLNAKLITQRKFDNLTKAERGGLSELDKAGFIKRQLVETRQITKHVAQILDSRMNTKYD
ENDKLIREVKVITLKSKLVSDFRKDFQFYKVREINNYHHAHDAYLNAVVGTALIKKYPKL
ESEFVYGDYKVYDVRKMIAKSEQEIGKATAKYFFYSNIMNFFKTEITLANGEIRKRPLIE
TNGETGEIVWDKGRDFATVRKVLSMPQVNIVKKTEVQTGGFSKESILPKRNSDKLIARKK
DWDPKKYGGFDSPTVAYSVLVVAKVEKGKSKKLKSVKELLGITIMERSSFEKNPIDFLEA
KGYKEVKKDLIIKLPKYSLFELENGRKRMLASAGELQKGNELALPSKYVNFLYLASHYEK
LKGSPEDNEQKQLFVEQHKHYLDEIIEQISEFSKRVILADANLDKVLSAYNKHRDKPIRE
QAENIIHLFTLTNLGAPAAFKYFDTTIDRKRYTSTKEVLDATLIHQSITGLYETRIDLSQ
LGGDEGAPKKKRKVGSSGSPKKKRKVGSDALDDFDLDMLGSDALDDFDLDMLGSDALDDF
DLDMLGSDALDDFDLDMLGSPKKKRKVGSSELIKENMHMKLYMEGTVDNHHFKCTSEGEG
KPYEGTQTMRIKVVEGGPLPFAFDILATSFLYGSKTFINHTQGIPDFFKQSFPEGFTWER
VTTYEDGGVLTATQDTSLQDGCLIYNVKIRGVNFTSNGPVMQKKTLGWEAFTETLYPADG
GLEGRNDMALKLVGGSHLIANIKTTYRSKKPAKNLKMPGVYYVDYRLERIKEANNETYVE
QHEVAVARYCDLPSKLGHKLN

(**ii**) Split ERT-**A**-VP64:

N-dCas9 fragment:

Blue = HA tag; Green = Gly-Ser linker; Dark red = FKBP; Yellow = ERT

MDKKYSIGLAIGTNSVGWAVITDEYKVPSKKFKVLGNTDRHSIKKNLIGALLFDSGETAE
ATRLKRTARRRYTRRKNRICYLQEIFSNEMAKVDDSFFHRLEESFLVEEDKKHERHPIFG
NIVDEVAYHEKYPTIYHLRKKLVDSTDKADLRLIYLALAHMIKFRGHFLIEGDLGGGGSG
GGGSEMWHEGLEEASRLYFGERNVKGMFEVLEPLHAMMERGPQTLKETSFNQAYGRDLME
AQEWCRKYMKSGNVKDLTQAWDLYYHVFRRISKQGGSLEPSAGDMRAANLWPSPLMIKRS
KKNSLALSLTADQMVSALLDAEPPILYSEYDPTRPFSEASMMGLLTNLADRELVHMINWA
KRVPGFVDLTLHDQVHLLECAWLEILMIGLVWRSMEHPVKLLFAPNLLLDRNQGKCVEGM
VEIFDMLLATSSRFRMMNLQGEEFVCLKSIILLNSGVYTFLSSTLKSLEEKDHIHRVLDK
ITDTLIHLMAKAGLTLQQQHQRLAQLLLILSHIRHMSNKGMEHLYSMKCKNVVPLYDLLL
EAADAHRLHAPTSRGGASVEETDQSHLATAGSTSSHSLQKYYITGEAEGFPATAGGSYPY
DVPDYA

C-dCas9 fragment:

Blue = myc tag; Orange = NLS; Green = Gly-Ser linker; Dark red = FKBP; Yellow = ERT; Turquoise = VP64; Blue = BFP

MEQKLISEEDLLESAGDMRAANLWPSPLMIKRSKKNSLALSLTADQMVSALLDAEPPILY
SEYDPTRPFSEASMMGLLTNLADRELVHMINWAKRVPGFVDLTLHDQVHLLECAWLEILM
IGLVWRSMEHPVKLLFAPNLLLDRNQGKCVEGMVEIFDMLLATSSRFRMMNLQGEEFVCL
KSIILLNSGVYTFLSSTLKSLEEKDHIHRVLDKITDTLIHLMAKAGLTLQQQHQRLAQLL
LILSHIRHMSNKGMEHLYSMKCKNVVPLYDLLLEAADAHRLHAPTSRGGASVEETDQSHL
ATAGSTSSHSLQKYYITGEAEGFPATAGGGGSGGGGSGVQVETISPGDGRTFPKRGQTCV

VHYTGMLEDGKKFDSSRDRNKPFKFMLGKQEVIRGWEEGVAQMSVGQRAKLTISPDYAYG
ATGHPGIIPPHATLVFDVELLKLEGGGGSGGGGSNPDNSDVDKLFIQLVQTYNQLFEENP
INASGVDAKAILSARLSKSRRLENLIAQLPGEKKNGLFGNLIALSLGLTPNFKSNFDLAE
DAKLQLSKDTYDDDLDNLLAQIGDQYADLFLAAKNLSDAILLSDILRVNTEITKAPLSAS
MIKRYDEHHQDLTLLKALVRQQLPEKYKEIFFDQSKNGYAGYIDGGASQEEFYKFIKPIL
EKMDGTEELLVKLNREDLLRKQRTFDNGSIPHQIHLGELHAILRRQEDFYPFLKDNREKI
EKILTFRIPYYVGPLARGNSRFAWMTRKSEETITPWNFEEVVDKGASAQSFIERMTNFDK
NLPNEKVLPKHSLLYEYFTVYNELTKVKYVTEGMRKPAFLSGEQKKAIVDLLFKTNRKVT
VKQLKEDYFKKIECFDSVEISGVEDRFNASLGTYHDLLKIIKDKDFLDNEENEDILEDIV
LTLTLFEDREMIEERLKTYAHLFDDKVMKQLKRRRYTGWGRLSRKLINGIRDKQSGKTIL
DFLKSDGFANRNFMQLIHDDSLTFKEDIQKAQVSGQGDSLHEHIANLAGSPAIKKGILQT
VKVVDELVKVMGRHKPENIVIEMARENQTTQKGQKNSRERMKRIEEGIKELGSQILKEHP
VENTQLQNEKLYLYYLQNGRDMYVDQELDINRLSDYDVDAIVPQSFLKDDSIDNKVLTRS
DKNRGKSDNVPSEEVVKKMKNYWRQLLNAKLITQRKFDNLTKAERGGLSELDKAGFIKRQ
LVETRQITKHVAQILDSRMNTKYDENDKLIREVKVITLKSKLVSDFRKDFQFYKVREINN
YHHAHDAYLNAVVGTALIKKYPKLESEFVYGDYKVYDVRKMIAKSEQEIGKATAKYFFYS
NIMNFFKTEITLANGEIRKRPLIETNGETGEIVWDKGRDFATVRKVLSMPQVNIVKKTEV
QTGGFSKESILPKRNSDKLIARKKDWDPKKYGGFDSPTVAYSVLVVAKVEKGKSKKLKSV
KELLGITIMERSSFEKNPIDFLEAKGYKEVKKDLIIKLPKYSLFELENGRKRMLASAGEL
QKGNELALPSKYVNFLYLASHYEKLKGSPEDNEQKQLFVEQHKHYLDEIIEQISEFSKRV
ILADANLDKVLSAYNKHRDKPIREQAENIIHLFTLTNLGAPAAFKYFDTTIDRKRYTSTK
EVLDATLIHQSITGLYETRIDLSQLGGDEGAPKKKRKVGSSGSPKKKRKVGSDALDDFDL
DMLGSDALDDFDLDMLGSDALDDFDLDMLGSDALDDFDLDMLGSPKKKRKVGS[redacted]

(**iii**) Split ERT-**A**-VPR:

N-dCas9 fragment:

Blue = HA tag; Green = Gly-Ser linker; Dark red = FKBP; Yellow = ERT

MDKKYSIGLAIGTNSVGWAVITDEYKVPSKKFKVLGNTDRHSIKKNLIGALLFDSGETAE
ATRLKRTARRRYTRRKNRICYLQEIFSNEMAKVDDSFFHRLEESFLVEEDKKHERHPIFG
NIVDEVAYHEKYPTIYHLRKKLVDSTDKADLRLIYLALAHMIKFRGHFLIEGDLGGGGSG
GGGSEMWHEGLEEASRLYFGERNVKGMFEVLEPLHAMMERGPQTLKETSFNQAYGRDLME
AQEWCRKYMKSGNVKDLTQAWDLYYHVFRRISKQGGSLEPSAGDMRAANLWPSPLMIKRS
KKNSLALSLTADQMVSALLDAEPPILYSEYDPTRPFSEASMMGLLTNLADRELVHMINWA
KRVPGFVDLTLHDQVHLLECAWLEILMIGLVWRSMEHPVKLLFAPNLLLDRNQGKCVEGM
VEIFDMLLATSSRFRMMNLQGEEFVCLKSIILLNSGVYTFLSSTLKSLEEKDHIHRVLDK
ITDTLIHLMAKAGLTLQQQHQRLAQLLLILSHIRHMSNKGMEHLYSMKCKNVVPLYDLLL
EAADAHRLHAPTSRGGASVEETDQSHLATAGSTSSHSLQKYYITGEAEGFPATAGGSYPY
DVPDYA

C-Cas9 fragment:

Blue = myc tag; Orange = NLS; Green = Gly-Ser linker; Dark red = FKBP; Yellow = ERT; Turquoise = VPR

MEQKLISEEDLLESAGDMRAANLWPSPLMIKRSKKNSLALSLTADQMVSALLDAEPPILY
SEYDPTRPFSEASMMGLLTNLADRELVHMINWAKRVPGFVDLTLHDQVHLLECAWLEILM
IGLVWRSMEHPVKLLFAPNLLLDRNQGKCVEGMVEIFDMLLATSSRFRMMNLQGEEFVCL
KSIILLNSGVYTFLSSTLKSLEEKDHIHRVLDKITDTLIHLMAKAGLTLQQQHQRLAQLL
LILSHIRHMSNKGMEHLYSMKCKNVVPLYDLLLEAADAHRLHAPTSRGGASVEETDQSHL
ATAGSTSSHSLQKYYITGEAEGFPATAGGGGSGGGGSGVQVETISPGDGRTFPKRGQTCV
VHYTGMLEDGKKFDSSRDRNKPFKFMLGKQEVIRGWEEGVAQMSVGQRAKLTISPDYAYG
ATGHPGIIPPHATLVFDVELLKLEGGGGSGGGGSNPDNSDVDKLFIQLVQTYNQLFEENP
INASGVDAKAILSARLSKSRRLENLIAQLPGEKKNGLFGNLIALSLGLTPNFKSNFDLAE
DAKLQLSKDTYDDDLDNLLAQIGDQYADLFLAAKNLSDAILLSDILRVNTEITKAPLSAS
MIKRYDEHHQDLTLLKALVRQQLPEKYKEIFFDQSKNGYAGYIDGGASQEEFYKFIKPIL
EKMDGTEELLVKLNREDLLRKQRTFDNGSIPHQIHLGELHAILRRQEDFYPFLKDNREKI

```
EKILTFRIPYYVGPLARGNSRFAWMTRKSEETITPWNFEEVVDKGASAQSFIERMTNFDK
NLPNEKVLPKHSLLYEYFTVYNELTKVKYVTEGMRKPAFLSGEQKKAIVDLLFKTNRKVT
VKQLKEDYFKKIECFDSVEISGVEDRFNASLGTYHDLLKIIKDKDFLDNEENEDILEDIV
LTLTLFEDREMIEERLKTYAHLFDDKVMKQLKRRRYTGWGRLSRKLINGIRDKQSGKTIL
DFLKSDGFANRNFMQLIHDDSLTFKEDIQKAQVSGQGDSLHEHIANLAGSPAIKKGILQT
VKVVDELVKVMGRHKPENIVIEMARENQTTQKGQKNSRERMKRIEEGIKELGSQILKEHP
VENTQLQNEKLYLYYLQNGRDMYVDQELDINRLSDYDVAAIVPQSFLKDDSIDNKVLTRS
DKARGKSDNVPSEEVVKKMKNYWRQLLNAKLITQRKFDNLTKAERGGLSELDKAGFIKRQ
LVETRQITKHVAQILDSRMNTKYDENDKLIREVKVITLKSKLVSDFRKDFQFYKVREINN
YHHAHDAYLNAVVGTALIKKYPKLESEFVYGDYKVYDVRKMIAKSEQEIGKATAKYFFYS
NIMNFFKTEITLANGEIRKRPLIETNGETGEIVWDKGRDFATVRKVLSMPQVNIVKKTEV
QTGGFSKESILPKRNSDKLIARKKDWDPKKYGGFDSPTVAYSVLVVAKVEKGKSKKLKSV
KELLGITIMERSSFEKNPIDFLEAKGYKEVKKDLIIKLPKYSLFELENGRKRMLASAGEL
QKGNELALPSKYVNFLYLASHYEKLKGSPEDNEQKQLFVEQHKHYLDEIIEQISEFSKRV
ILADANLDKVLSAYNKHRDKPIREQAENIIHLFTLTNLGAPAAFKYFDTTIDRKRYTSTK
EVLDATLIHQSITGLYETRIDLSQLGGDSRADPKKKRKVSPGIRRLDALISTSLYKKAGY
KEASGSGRADALDDFDLDMLGSDALDDFDLDMLGSDALDDFDLDMLGSDALDDFDLDMLI
NSRSSGSPKKKRKVGSQYLPDTDDRHRIEEKRKRTYETFKSIMKKSPFSGPTDPRPPPRR
IAVPSRSSASVPKPAPQPYPFTSSLSTINYDEFPTMVFPSGQISQASALAPAPPQVLPQA
PAPAPAPAMVSALAQAPAPVPVLAPGPPQAVAPPAPKPTQAGEGTLSEALLQLQFDDEDL
GALLGNSTDPAVFTDLASVDNSEFQQLLNQGIPVAPHTTEPMLMEYPEAITRLVTGAQRP
PDPAPAPLGAPGLPNGLLSGDEDFSSIADMDFSALLGSGSGSRDSREGMFLPKPEAGSAI
SDVFEGREVCQPKRIRPFHPPGSPWANRPLPASLAPTPTGPVHEPVGSLTPAPVPQPLDP
APAVTPEASHLLEDPDEETSQAVKALREMADTVIPQKEEAAICGQMDLSHPPPRGHLDEL
TTTLESMTEDLNLDSPLTPELNEILDTFLNDECLLHAMHISTGLSIFDTSLF
```

(**iv**) Split ERT-**C**-VPR:

N-dCas9 fragment:

Blue = HA tag; Green = Gly-Ser linker; Dark red = FKBP; Yellow = ERT

```
MDKKYSIGLAIGTNSVGWAVITDEYKVPSKKFKVLGNTDRHSIKKNLIGALLFDSGETAE
ATRLKRTARRRYTRRKNRICYLQEIFSNEMAKVDDSFFHRLEESFLVEEDKKHERHPIFG
NIVDEVAYHEKYPTIYHLRKKLVDSTDKADLRLIYLALAHMIKFRGHFLIEGDLNPDNSD
VDKLFIQLVQTYNQLFEENPINASGGGGSGGGGSEMWHEGLEEASRLYFGERNVKGMFEV
LEPLHAMMERGPQTLKETSFNQAYGRDLMEAQEWCRKYMKSGNVKDLTQAWDLYYHVFRR
ISKQGGSLEPSAGDMRAANLWPSPLMIKRSKKNSLALSLTADQMVSALLDAEPPILYSEY
DPTRPFSEASMMGLLTNLADRELVHMINWAKRVPGFVDLTLHDQVHLLECAWLEILMIGL
VWRSMEHPVKLLFAPNLLLDRNQGKCVEGMVEIFDMLLATSSRFRMMNLQGEEFVCLKSI
ILLNSGVYTFLSSTLKSLEEKDHIHRVLDKITDTLIHLMAKAGLTLQQQHQRLAQLLLIL
SHIRHMSNKGMEHLYSMKCKNVVPLYDLLLEAADAHRLHAPTSRGGASVEETDQSHLATA
GSTSSHSLQKYYITGEAEGFPATAGGSYPYDVPDYA
```

C-Cas9 fragment:

Blue = myc tag; Orange = NLS; Green = Gly-Ser linker; Dark red = FKBP; Yellow = ERT; Turquoise = VPR

```
MEQKLISEEDLLESAGDMRAANLWPSPLMIKRSKKNSLALSLTADQMVSALLDAEPPILY
SEYDPTRPFSEASMMGLLTNLADRELVHMINWAKRVPGFVDLTLHDQVHLLECAWLEILM
IGLVWRSMEHPVKLLFAPNLLLDRNQGKCVEGMVEIFDMLLATSSRFRMMNLQGEEFVCL
KSIILLNSGVYTFLSSTLKSLEEKDHIHRVLDKITDTLIHLMAKAGLTLQQQHQRLAQLL
LILSHIRHMSNKGMEHLYSMKCKNVVPLYDLLLEAADAHRLHAPTSRGGASVEETDQSHL
ATAGSTSSHSLQKYYITGEAEGFPATAGGGGSGGGGSGVQVETISPGDGRTFPKRGQTCV
VHYTGMLEDGKKFDSSRDRNKPFKFMLGKQEVIRGWEEGVAQMSVGQRAKLTISPDYAYG
ATGHPGIIPPHATLVFDVELLKLEGGGGSGGGGSGVDAKAILSARLSKSRRLENLIAQLP
GEKKNGLFGNLIALSLGLTPNFKSNFDLAEDAKLQLSKDTYDDDLDNLLAQIGDQYADLF
LAAKNLSDAILLSDILRVNTEITKAPLSASMIKRYDEHHQDLTLLKALVRQQLPEKYKEI
FFDQSKNGYAGYIDGGASQEEFYKFIKPILEKMDGTEELLVKLNREDLLRKQRTFDNGSI
PHQIHLGELHAILRRQEDFYPFLKDNREKIEKILTFRIPYYVGPLARGNSRFAWMTRKSE
ETITPWNFEEVVDKGASAQSFIERMTNFDKNLPNEKVLPKHSLLYEYFTVYNELTKVKYV
```

TEGMRKPAFLSGEQKKAIVDLLFKTNRKVTVKQLKEDYFKKIECFDSVEISGVEDRFNAS
LGTYHDLLKIIKDKDFLDNEENEDILEDIVLTLTLFEDREMIEERLKTYAHLFDDKVMKQ
LKRRRYTGWGRLSRKLINGIRDKQSGKTILDFLKSDGFANRNFMQLIHDDSLTFKEDIQK
AQVSGQGDSLHEHIANLAGSPAIKKGILQTVKVVDELVKVMGRHKPENIVIEMARENQTT
QKGQKNSRERMKRIEEGIKELGSQILKEHPVENTQLQNEKLYLYYLQNGRDMYVDQELDI
NRLSDYDVAAIVPQSFLKDDSIDNKVLTRSDKARGKSDNVPSEEVVKKMKNYWRQLLNAK
LITQRKFDNLTKAERGGLSELDKAGFIKRQLVETRQITKHVAQILDSRMNTKYDENDKLI
REVKVITLKSKLVSDFRKDFQFYKVREINNYHHAHDAYLNAVVGTALIKKYPKLESEFVY
GDYKVYDVRKMIAKSEQEIGKATAKYFFYSNIMNFFKTEITLANGEIRKRPLIETNGETG
EIVWDKGRDFATVRKVLSMPQVNIVKKTEVQTGGFSKESILPKRNSDKLIARKKDWDPKK
YGGFDSPTVAYSVLVVAKVEKGKSKKLKSVKELLGITIMERSSFEKNPIDFLEAKGYKEV
KKDLIIKLPKYSLFELENGRKRMLASAGELQKGNELALPSKYVNFLYLASHYEKLKGSPE
DNEQKQLFVEQHKHYLDEIIEQISEFSKRVILADANLDKVLSAYNKHRDKPIREQAENII
HLFTLTNLGAPAAFKYFDTTIDRKRYTSTKEVLDATLIHQSITGLYETRIDLSQLGGDSR
ADPKKKRKVSPGIRRLDALISTSLYKKAGYKEASGSGRADALDDFDLDMLGSDALDDFDL
DMLGSDALDDFDLDMLGSDALDDFDLDMLINSRSSGSPKKKRKVGSQYLPDTDDRHRIEE
KRKRTYETFKSIMKKSPFSGPTDPRPPPRRIAVPSRSSASVPKPAPQPYPFTSSLSTINY
DEFPTMVFPSGQISQASALAPAPPQVLPQAPAPAPAPAMVSALAQAPAPVPVLAPGPPQA
VAPPAPKPTQAGEGTLSEALLQLQFDDEDLGALLGNSTDPAVFTDLASVDNSEFQQLLNQ
GIPVAPHTTEPMLMEYPEAITRLVTGAQRPPDPAPAPLGAPGLPNGLLSGDEDFSSIADM
DFSALLGSGSGSRDSREGMFLPKPEAGSAISDVFEGREVCQPKRIRPFHPPGSPWANRPL
PASLAPTPTGPVHEPVGSLTPAPVPQPLDPAPAVTPEASHLLEDPDEETSQAVKALREMA
DTVIPQKEEAAICGQMDLSHPPPRGHLDELTTTLESMTEDLNLDSPLTPELNEILDTFLN
DECLLHAMHISTGLSIFDTSLF

(**v**) Split ERT-**A**-VPR$^{\Delta NLS}$:

N-dCas9 fragment:

Blue = HA tag; Green = Gly-Ser linker; Dark red = FKBP; Yellow = ERT

MDKKYSIGLAIGTNSVGWAVITDEYKVPSKKFKVLGNTDRHSIKKNLIGALLFDSGETAE
ATRLKRTARRRYTRRKNRICYLQEIFSNEMAKVDDSFFHRLEESFLVEEDKKHERHPIFG
NIVDEVAYHEKYPTIYHLRKKLVDSTDKADLRLIYLALAHMIKFRGHFLIEGDLGGGGSG
GGGSEMWHEGLEEASRLYFGERNVKGMFEVLEPLHAMMERGPQTLKETSFNQAYGRDLME
AQEWCRKYMKSGNVKDLTQAWDLYYHVFRRISKQGGSLEPSAGDMRAANLWPSPLMIKRS
KKNSLALSLTADQMVSALLDAEPPILYSEYDPTRPFSEASMMGLLTNLADRELVHMINWA
KRVPGFVDLTLHDQVHLLECAWLEILMIGLVWRSMEHPVKLLFAPNLLLDRNQGKCVEGM
VEIFDMLLATSSRFRMMNLQGEEFVCLKSIILLNSGVYTFLSSTLKSLEEKDHIHRVLDK
ITDTLIHLMAKAGLTLQQQHQRLAQLLLILSHIRHMSNKGMEHLYSMKCKNVVPLYDLLL
EAADAHRLHAPTSRGGASVEETDQSHLATAGSTSSHSLQKYYITGEAEGFPATAGGSYPY
DVPDYA

C-Cas9 fragment:

Blue = myc tag; Green = Gly-Ser linker; Dark red = FKBP; Yellow = ERT; Turquoise = VPR$^{\Delta NLS}$

MEQKLISEEDLLESAGDMRAANLWPSPLMIKRSKKNSLALSLTADQMVSALLDAEPPILY
SEYDPTRPFSEASMMGLLTNLADRELVHMINWAKRVPGFVDLTLHDQVHLLECAWLEILM
IGLVWRSMEHPVKLLFAPNLLLDRNQGKCVEGMVEIFDMLLATSSRFRMMNLQGEEFVCL
KSIILLNSGVYTFLSSTLKSLEEKDHIHRVLDKITDTLIHLMAKAGLTLQQQHQRLAQLL
LILSHIRHMSNKGMEHLYSMKCKNVVPLYDLLLEAADAHRLHAPTSRGGASVEETDQSHL
ATAGSTSSHSLQKYYITGEAEGFPATAGGGGSGGGGSGVQVETISPGDGRTFPKRGQTCV
VHYTGMLEDGKKFDSSRDRNKPFKFMLGKQEVIRGWEEGVAQMSVGQRAKLTISPDYAYG
ATGHPGIIPPHATLVFDVELLKLEGGGGSGGGGSNPDNSDVDKLFIQLVQTYNQLFEENP
INASGVDAKAILSARLSKSRRLENLIAQLPGEKKNGLFGNLIALSLGLTPNFKSNFDLAE
DAKLQLSKDTYDDDLDNLLAQIGDQYADLFLAAKNLSDAILLSDILRVNTEITKAPLSAS
MIKRYDEHHQDLTLLKALVRQQLPEKYKEIFFDQSKNGYAGYIDGGASQEEFYKFIKPIL
EKMDGTEELLVKLNREDLLRKQRTFDNGSIPHQIHLGELHAILRRQEDFYPFLKDNREKI
EKILTFRIPYYVGPLARGNSRFAWMTRKSEETITPWNFEEVVDKGASAQSFIERMTNFDK
NLPNEKVLPKHSLLYEYFTVYNELTKVKYVTEGMRKPAFLSGEQKKAIVDLLFKTNRKVT

```
VKQLKEDYFKKIECFDSVEISGVEDRFNASLGTYHDLLKIIKDKDFLDNEENEDILEDIV
LTLTLFEDREMIEERLKTYAHLFDDKVMKQLKRRRYTGWGRLSRKLINGIRDKQSGKTIL
DFLKSDGFANRNFMQLIHDDSLTFKEDIQKAQVSGQGDSLHEHIANLAGSPAIKKGILQT
VKVVDELVKVMGRHKPENIVIEMARENQTTQKGQKNSRERMKRIEEGIKELGSQILKEHP
VENTQLQNEKLYLYYLQNGRDMYVDQELDINRLSDYDVAAIVPQSFLKDDSIDNKVLTRS
DKARGKSDNVPSEEVVKKMKNYWRQLLNAKLITQRKFDNLTKAERGGLSELDKAGFIKRQ
LVETRQITKHVAQILDSRMNTKYDENDKLIREVKVITLKSKLVSDFRKDFQFYKVREINN
YHHAHDAYLNAVVGTALIKKYPKLESEFVYGDYKVYDVRKMIAKSEQEIGKATAKYFFYS
NIMNFFKTEITLANGEIRKRPLIETNGETGEIVWDKGRDFATVRKVLSMPQVNIVKKTEV
QTGGFSKESILPKRNSDKLIARKKDWDPKKYGGFDSPTVAYSVLVVAKVEKGKSKKLKSV
KELLGITIMERSSFEKNPIDFLEAKGYKEVKKDLIIKLPKYSLFELENGRKRMLASAGEL
QKGNELALPSKYVNFLYLASHYEKLKGSPEDNEQKQLFVEQHKHYLDEIIEQISEFSKRV
ILADANLDKVLSAYNKHRDKPIREQAENIIHLFTLTNLGAPAAFKYFDTTIDRKRYTSTK
EVLDATLIHQSITGLYETRIDLSQLGGDSRADSPGIRRLDALISTSLYKKAGYKEASGSG
RADALDDFDLDMLGSDALDDFDLDMLGSDALDDFDLDMLGSDALDDFDLDMLINSRGGSQ
YLPDTDDRHRIEEKRKRTYETFKSIMKKSPFSGPTDPRPPPRRIAVPSRSSASVPKPAPQ
PYPFTSSLSTINYDEFPTMVFPSGQISQASALAPAPPQVLPQAPAPAPAPAMVSALAQAP
APVPVLAPGPPQAVAPPAPKPTQAGEGTLSEALLQLQFDDEDLGALLGNSTDPAVFTDLA
SVDNSEFQQLLNQGIPVAPHTTEPMLMEYPEAITRLVTGAQRPPDPAPAPLGAPGLPNGL
LSGDEDFSSIADMDFSALLGSGSGSRDSREGMFLPKPEAGSAISDVFEGREVCQPKRIRP
FHPPGSPWANRPLPASLAPTPTGPVHEPVGSLTPAPVPQPLDPAPAVTPEASHLLEDPDE
ETSQAVKALREMADTVIPQKEEAAICGQMDLSHPPPRGHLDELTTTLESMTEDLNLDSPL
TPELNEILDTFLNDECLLHAMHISTGLSIFDTSLF
```

(iv) Split GR-**A**-VPR:

N-dCas9 fragment:

Blue = HA tag; Green = Gly-Ser linker; Dark red = FKBP; Yellow = GR

```
MYPYDVPDYASPKKKRKVEASDKKYSIGLAIGTNSVGWAVITDEYKVPSKKFKVLGNTDR
HSIKKNLIGALLFDSGETAEATRLKRTARRRYTRRKNRICYLQEIFSNEMAKVDDSFFHR
LEESFLVEEDKKHERHPIFGNIVDEVAYHEKYPTIYHLRKKLVDSTDKADLRLIYLALAH
MIKFRGHFLIEGDLGGGGSGGGGSEMWHEGLEEASRLYFGERNVKGMFEVLEPLHAMMER
GPQTLKETSFNQAYGRDLMEAQEWCRKYMKSGNVKDLTQAWDLYYHVFRRISKQGGSVPA
TLPQLTPTLVSLLEVIEPEVLYAGYDSSVPDSTWRIMTTLNMLGGRQVIAAVKWAKAIPG
FRNLHLDDQMTLLQYSWMFLMAFALGWRSYRQSSANLLCFAPDLIINEQRMTLPCMYDQC
KHMLYVSSELHRLQVSYEEYLCMKTLLLLSSVPKDGLKSQELFDEIRMTYIKELGKAIVK
REGNSSQNWQRFYQLTKLLDSMHEVVENLLNYCFQTFLDKTMSIEFPEMLAEIITNQIPK
YSNGNIKKLLFHQK
```

C-dCas9 fragment:

Blue = myc tag; Orange = NLS; Green = Gly-Ser linker; Dark red = FKBP; Yellow = GR; Turquoise = VPR

```
MEQKLISEEDLLEVPATLPQLTPTLVSLLEVIEPEVLYAGYDSSVPDSTWRIMTTLNMLG
GRQVIAAVKWAKAIPGFRNLHLDDQMTLLQYSWMFLMAFALGWRSYRQSSANLLCFAPDL
IINEQRMTLPCMYDQCKHMLYVSSELHRLQVSYEEYLCMKTLLLLSSVPKDGLKSQELFD
EIRMTYIKELGKAIVKREGNSSQNWQRFYQLTKLLDSMHEVVENLLNYCFQTFLDKTMSI
EFPEMLAEIITNQIPKYSNGNIKKLLFHQKGGSGVQVETISPGDGRTFPKRGQTCVVHYT
GMLEDGKKFDSSRDRNKPFKFMLGKQEVIRGWEEGVAQMSVGQRAKLTISPDYAYGATGH
PGIIPPHATLVFDVELLKLEGGGGSGGGGSNPDNSDVDKLFIQLVQTYNQLFEENPINAS
GVDAKAILSARLSKSRRLENLIAQLPGEKKNGLFGNLIALSLGLTPNFKSNFDLAEDAKL
QLSKDTYDDDLDNLLAQIGDQYADLFLAAKNLSDAILLSDILRVNTEITKAPLSASMIKR
YDEHHQDLTLLKALVRQQLPEKYKEIFFDQSKNGYAGYIDGGASQEEFYKFIKPILEKMD
GTEELLVKLNREDLLRKQRTFDNGSIPHQIHLGELHAILRRQEDFYPFLKDNREKIEKIL
TFRIPYYVGPLARGNSRFAWMTRKSEETITPWNFEEVVDKGASAQSFIERMTNFDKNLPN
EKVLPKHSLLYEYFTVYNELTKVKYVTEGMRKPAFLSGEQKKAIVDLLFKTNRKVTVKQL
KEDYFKKIECFDSVEISGVEDRFNASLGTYHDLLKIIKDKDFLDNEENEDILEDIVLTLT
LFEDREMIEERLKTYAHLFDDKVMKQLKRRRYTGWGRLSRKLINGIRDKQSGKTILDFLK
SDGFANRNFMQLIHDDSLTFKEDIQKAQVSGQGDSLHEHIANLAGSPAIKKGILQTVKVV
DELVKVMGRHKPENIVIEMARENQTTQKGQKNSRERMKRIEEGIKELGSQILKEHPVENT
```

QLQNEKLYLYYLQNGRDMYVDQELDINRLSDYDVDAIVPQSFLKDDSIDNKVLTRSDKNR
GKSDNVPSEEVVKKMKNYWRQLLNAKLITQRKFDNLTKAERGGLSELDKAGFIKRQLVET
RQITKHVAQILDSRMNTKYDENDKLIREVKVITLKSKLVSDFRKDFQFYKVREINNYHHA
HDAYLNAVVGTALIKKYPKLESEFVYGDYKVYDVRKMIAKSEQEIGKATAKYFFYSNIMN
FFKTEITLANGEIRKRPLIETNGETGEIVWDKGRDFATVRKVLSMPQVNIVKKTEVQTGG
FSKESILPKRNSDKLIARKKDWDPKKYGGFDSPTVAYSVLVVAKVEKGKSKKLKSVKELL
GITIMERSSFEKNPIDFLEAKGYKEVKKDLIIKLPKYSLFELENGRKRMLASAGELQKGN
ELALPSKYVNFLYLASHYEKLKGSPEDNEQKQLFVEQHKHYLDEIIEQISEFSKRVILAD
ANLDKVLSAYNKHRDKPIREQAENIIHLFTLTNLGAPAAFKYFDTTIDRKRYTSTKEVLD
ATLIHQSITGLYETRIDLSQLGGDSRADPKKKRKVSPGIRRLDALISTSLYKKAGYKEAS
GSGRADALDDFDLDMLGSDALDDFDLDMLGSDALDDFDLDMLGSDALDDFDLDMLINSRS
SGSPKKKRKVGSQYLPDTDDRHRIEEKRKRTYETFKSIMKKSPFSGPTDPRPPPRRIAVP
SRSSASVPKPAPQPYPFTSSLSTINYDEFPTMVFPSGQISQASALAPAPPQVLPQAPAPA
PAPAMVSALAQAPAPVPVLAPGPPQAVAPPAPKPTQAGEGTLSEALLQLQFDDEDLGALL
GNSTDPAVFTDLASVDNSEFQQLLNQGIPVAPHTTEPMLMEYPEAITRLVTGAQRPPDPA
PAPLGAPGLPNGLLSGDEDFSSIADMDFSALLGSGSGSRDSREGMFLPKPEAGSAISDVF
EGREVCQPKRIRPFHPPGSPWANRPLPASLAPTPTGPVHEPVGSLTPAPVPQPLDPAPAV
TPEASHLLEDPDEETSQAVKALREMADTVIPQKEEAAICGQMDLSHPPPRGHLDELTTTL
ESMTEDLNLDSPLTPELNEILDTFLNDECLLHAMHISTGLSIFDTSLF

(**C**) Amino acid sequence of ERT-dCas9-VPR:

Blue = HA tag; Orange = NLS; Green = Gly-Ser linker; Yellow = ERT; Turquoise = VPR

MSAGDMRAANLWPSPLMIKRSKKNSLALSLTADQMVSALLDAEPPILYSEYDPTRPFSEA
SMMGLLTNLADRELVHMINWAKRVPGFVDLTLHDQVHLLECAWLEILMIGLVWRSMEHPV
KLLFAPNLLLDRNQGKCVEGMVEIFDMLLATSSRFRMMNLQGEEFVCLKSIILLNSGVYT
FLSSTLKSLEEKDHIHRVLDKITDTLIHLMAKAGLTLQQQHQRLAQLLLILSHIRHMSNK
GMEHLYSMKCKNVVPLYDLLLEAADAHRLHAPTSRGGASVEETDQSHLATAGSTSSHSLQ
KYYITGEAEGFPATAGGGGSGGGGSYPYDVPDYASPKKKRKVEASDKKYSIGLAIGTNSV
GWAVITDEYKVPSKKFKVLGNTDRHSIKKNLIGALLFDSGETAEATRLKRTARRRYTRRK
NRICYLQEIFSNEMAKVDDSFFHRLEESFLVEEDKKHERHPIFGNIVDEVAYHEKYPTIY
HLRKKLVDSTDKADLRLIYLALAHMIKFRGHFLIEGDLNPDNSDVDKLFIQLVQTYNQLF
EENPINASGVDAKAILSARLSKSRRLENLIAQLPGEKKNGLFGNLIALSLGLTPNFKSNF
DLAEDAKLQLSKDTYDDDLDNLLAQIGDQYADLFLAAKNLSDAILLSDILRVNTEITKAP
LSASMIKRYDEHHQDLTLLKALVRQQLPEKYKEIFFDQSKNGYAGYIDGGASQEEFYKFI
KPILEKMDGTEELLVKLNREDLLRKQRTFDNGSIPHQIHLGELHAILRRQEDFYPFLKDN
REKIEKILTFRIPYYVGPLARGNSRFAWMTRKSEETITPWNFEEVVDKGASAQSFIERMT
NFDKNLPNEKVLPKHSLLYEYFTVYNELTKVKYVTEGMRKPAFLSGEQKKAIVDLLFKTN
RKVTVKQLKEDYFKKIECFDSVEISGVEDRFNASLGTYHDLLKIIKDKDFLDNEENEDIL
EDIVLTLTLFEDREMIEERLKTYAHLFDDKVMKQLKRRRYTGWGRLSRKLINGIRDKQSG
KTILDFLKSDGFANRNFMQLIHDDSLTFKEDIQKAQVSGQGDSLHEHIANLAGSPAIKKG
ILQTVKVVDELVKVMGRHKPENIVIEMARENQTTQKGQKNSRERMKRIEEGIKELGSQIL
KEHPVENTQLQNEKLYLYYLQNGRDMYVDQELDINRLSDYDVDAIVPQSFLKDDSIDNKV
LTRSDKNRGKSDNVPSEEVVKKMKNYWRQLLNAKLITQRKFDNLTKAERGGLSELDKAGF
IKRQLVETRQITKHVAQILDSRMNTKYDENDKLIREVKVITLKSKLVSDFRKDFQFYKVR
EINNYHHAHDAYLNAVVGTALIKKYPKLESEFVYGDYKVYDVRKMIAKSEQEIGKATAKY
FFYSNIMNFFKTEITLANGEIRKRPLIETNGETGEIVWDKGRDFATVRKVLSMPQVNIVK
KTEVQTGGFSKESILPKRNSDKLIARKKDWDPKKYGGFDSPTVAYSVLVVAKVEKGKSKK
LKSVKELLGITIMERSSFEKNPIDFLEAKGYKEVKKDLIIKLPKYSLFELENGRKRMLAS
AGELQKGNELALPSKYVNFLYLASHYEKLKGSPEDNEQKQLFVEQHKHYLDEIIEQISEF
SKRVILADANLDKVLSAYNKHRDKPIREQAENIIHLFTLTNLGAPAAFKYFDTTIDRKRY
TSTKEVLDATLIHQSITGLYETRIDLSQLGGDSRADPKKKRKVSPGIRRLDALISTSLYK
KAGYKEASGSGRADALDDFDLDMLGSDALDDFDLDMLGSDALDDFDLDMLGSDALDDFDL
DMLINSRSSGSPKKKRKVGSQYLPDTDDRHRIEEKRKRTYETFKSIMKKSPFSGPTDPRP
PPRRIAVPSRSSASVPKPAPQPYPFTSSLSTINYDEFPTMVFPSGQISQASALAPAPPQV
LPQAPAPAPAPAMVSALAQAPAPVPVLAPGPPQAVAPPAPKPTQAGEGTLSEALLQLQFD
DEDLGALLGNSTDPAVFTDLASVDNSEFQQLLNQGIPVAPHTTEPMLMEYPEAITRLVTG
AQRPPDPAPAPLGAPGLPNGLLSGDEDFSSIADMDFSALLGSGSGSRDSREGMFLPKPEA
GSAISDVFEGREVCQPKRIRPFHPPGSPWANRPLPASLAPTPTGPVHEPVGSLTPAPVPQ
PLDPAPAVTPEASHLLEDPDEETSQAVKALREMADTVIPQKEEAAICGQMDLSHPPPRGH
LDELTTTLESMTEDLNLDSPLTPELNEILDTFLNDECLLHAMHISTGLSIFDTSLF

(**D**) Amino acid sequence of ERT-dCas9-VPR$^{\Delta NLS}$:

Blue = myc tag; Green = Gly-Ser linker; Yellow = ERT; Turquoise = VPR

```
MEQKLISEEDLLEVPATLPQLTPTLVSLLEVIEPEVLYAGYDSSVPDSTWRIMTTLNMLG
GRQVIAAVKWAKAIPGFRNLHLDDQMTLLQYSWMFLMAFALGWRSYRQSSANLLCFAPDL
IINEQRMTLPCMYDQCKHMLYVSSELHRLQVSYEEYLCMKTLLLLSSVPKDGLKSQELFD
EIRMTYIKELGKAIVKREGNSSQNWQRFYQLTKLLDSMHEVVENLLNYCFQTFLDKTMSI
EFPEMLAEIITNQIPKYSNGNIKKLLFHQKGGGGSGGGGSGGGGSDKKYSIGLAIGTNSV
GWAVITDEYKVPSKKFKVLGNTDRHSIKKNLIGALLFDSGETAEATRLKRTARRRYTRRK
NRICYLQEIFSNEMAKVDDSFFHRLEESFLVEEDKKHERHPIFGNIVDEVAYHEKYPTIY
HLRKKLVDSTDKADLRLIYLALAHMIKFRGHFLIEGDLNPDNSDVDKLFIQLVQTYNQLF
EENPINASGVDAKAILSARLSKSRRLENLIAQLPGEKKNGLFGNLIALSLGLTPNFKSNF
DLAEDAKLQLSKDTYDDDLDNLLAQIGDQYADLFLAAKNLSDAILLSDILRVNTEITKAP
LSASMIKRYDEHHQDLTLLKALVRQQLPEKYKEIFFDQSKNGYAGYIDGGASQEEFYKFI
KPILEKMDGTEELLVKLNREDLLRKQRTFDNGSIPHQIHLGELHAILRRQEDFYPFLKDN
REKIEKILTFRIPYYVGPLARGNSRFAWMTRKSEETITPWNFEEVVDKGASAQSFIERMT
NFDKNLPNEKVLPKHSLLYEYFTVYNELTKVKYVTEGMRKPAFLSGEQKKAIVDLLFKTN
RKVTVKQLKEDYFKKIECFDSVEISGVEDRFNASLGTYHDLLKIIKDKDFLDNEENEDIL
EDIVLTLTLFEDREMIEERLKTYAHLFDDKVMKQLKRRRYTGWGRLSRKLINGIRDKQSG
KTILDFLKSDGFANRNFMQLIHDDSLTFKEDIQKAQVSGQGDSLHEHIANLAGSPAIKKG
ILQTVKVVDELVKVMGRHKPENIVIEMARENQTTQKGQKNSRERMKRIEEGIKELGSQIL
KEHPVENTQLQNEKLYLYYLQNGRDMYVDQELDINRLSDYDVDAIVPQSFLKDDSIDNKV
LTRSDKNRGKSDNVPSEEVVKKMKNYWRQLLNAKLITQRKFDNLTKAERGGLSELDKAGF
IKRQLVETRQITKHVAQILDSRMNTKYDENDKLIREVKVITLKSKLVSDFRKDFQFYKVR
EINNYHHAHDAYLNAVVGTALIKKYPKLESEFVYGDYKVYDVRKMIAKSEQEIGKATAKY
FFYSNIMNFFKTEITLANGEIRKRPLIETNGETGEIVWDKGRDFATVRKVLSMPQVNIVK
KTEVQTGGFSKESILPKRNSDKLIARKKDWDPKKYGGFDSPTVAYSVLVVAKVEKGKSKK
LKSVKELLGITIMERSSFEKNPIDFLEAKGYKEVKKDLIIKLPKYSLFELENGRKRMLAS
AGELQKGNELALPSKYVNFLYLASHYEKLKGSPEDNEQKQLFVEQHKHYLDEIIEQISEF
SKRVILADANLDKVLSAYNKHRDKPIREQAENIIHLFTLTNLGAPAAFKYFDTTIDRKRY
TSTKEVLDATLIHQSITGLYETRIDLSQLGGDSRADSPGIRRLDALISTSLYKKAGYKEA
SGSGRADALDDFDLDMLGSDALDDFDLDMLGSDALDDFDLDMLGSDALDDFDLDMLINSR
GGSQYLPDTDDRHRIEEKRKRTYETFKSIMKKSPFSGPTDPRPPPRRIAVPSRSSASVPK
PAPQPYPFTSSLSTINYDEFPTMVFPSGQISQASALAPAPPQVLPQAPAPAPAPAMVSAL
AQAPAPVPVLAPGPPQAVAPPAPKPTQAGEGTLSEALLQLQFDDEDLGALLGNSTDPAVF
TDLASVDNSEFQQLLNQGIPVAPHTTEPMLMEYPEAITRLVTGAQRPPDPAPAPLGAPGL
PNGLLSGDEDFSSIADMDFSALLGSGSGSRDSREGMFLPKPEAGSAISDVFEGREVCQPK
RIRPFHPPGSPWANRPLPASLAPTPTGPVHEPVGSLTPAPVPQPLDPAPAVTPEASHLLE
DPDEETSQAVKALREMADTVIPQKEEAAICGQMDLSHPPPRGHLDELTTTLESMTEDLNL
DSPLTPELNEILDTFLNDECLLHAMHISTGLSIFDTSLF
```

(**E**) Amino acid sequence of C-fragment of pX-VPR:

Orange = NLS; Green = Gly-Ser linker; Dark red = FKBP12; Turquoise = VPR

```
MAPKKKRKVGIHGVPAAGVQVETISPGDGRTFPKRGQTCVVHYTGMLEDGKKFDSSRDRN
KPFKFMLGKQEVIRGWEEGVAQMSVGQRAKLTISPDYAYGATGHPGIIPPHATLVFDVEL
LKLEGGGGSGGGGSGGGGSKPAFLSGEQKKAIVDLLFKTNRKVTVKQLKEDYFKKIECFD
SVEISGVEDRFNASLGTYHDLLKIIKDKDFLDNEENEDILEDIVLTLTLFEDREMIEERL
KTYAHLFDDKVMKQLKRRRYTGWGRLSRKLINGIRDKQSGKTILDFLKSDGFANRNFMQL
IHDDSLTFKEDIQKAQVSGQGDSLHEHIANLAGSPAIKKGILQTVKVVDELVKVMGRHKP
ENIVIEMARENQTTQKGQKNSRERMKRIEEGIKELGSQILKEHPVENTQLQNEKLYLYYL
QNGRDMYVDQELDINRLSDYDVDHIVPQSFLKDDSIDNKVLTRSDKARGKSDNVPSEEVV
KKMKNYWRQLLNAKLITQRKFDNLTKAERGGLSELDKAGFIKRQLVETRQITKHVAQILD
SRMNTKYDENDKLIREVKVITLKSKLVSDFRKDFQFYKVREINNYHHAHDAYLNAVVGTA
LIKKYPKLESEFVYGDYKVYDVRKMIAKSEQEIGKATAKYFFYSNIMNFFKTEITLANGE
IRKRPLIETNGETGEIVWDKGRDFATVRKVLSMPQVNIVKKTEVQTGGFSKESILPKRNS
DKLIARKKDWDPKKYGGFDSPTVAYSVLVVAKVEKGKSKKLKSVKELLGITIMERSSFEK
NPIDFLEAKGYKEVKKDLIIKLPKYSLFELENGRKRMLASAGELQKGNELALPSKYVNFL
YLASHYEKLKGSPEDNEQKQLFVEQHKHYLDEIIEQISEFSKRVILADANLDKVLSAYNK
HRDKPIREQAENIIHLFTLTNLGAPAAFKYFDTTIDRKRYTSTKEVLDATLIHQSITGLY
```

ETRIDLSQLGGDSPKKKRKVEASGRADALDDFDLDMLGSDALDDFDLDMLGSDALDDFDL
DMLGSDALDDFDLDMLINSRGGSSQYLPDTDDRHRIEEKRKRTYETFKSIMKKSPFSGPT
DPRPPPRRIAVPSRSSASVPKPAPQPYPFTSSLSTINYDEFPTMVFPSGQISQASALAPA
PPQVLPQAPAPAPAPAMVSALAQAPAPVPVLAPGPPQAVAPPAPKPTQAGEGTLSEALLQ
LQFDDEDLGALLGNSTDPAVFTDLASVDNSEFQQLLNQGIPVAPHTTEPMLMEYPEAITR
LVTGAQRPPDPAPAPLGAPGLPNGLLSGDEDFSSIADMDFSALLGSGSGSRDSREGMFLP
KPEAGSAISDVFEGREVCQPKRIRPFHPPGSPWANRPLPASLAPTPTGPVHEPVGSLTPA
PVPQPLDPAPAVTPEASHLLEDPDEETSQAVKALREMADTVIPQKEEAAICGQMDLSHPP
PRGHLDELTTTLESMTEDLNLDSPLTPELNEILDTFLNDECLLHAMHISTGLSIFDTSLF

(**F**) Amino acid sequence of intein-VPR:

Orange = NLS; Blue = intein; Turquoise = VPR

MDKKYSIGLAIGTNSVGWAVITDEYKVPSKKFKVLGNTDRHSIKKNLIGALLFDSGETAE
ATRLKRTARRRYTRRKNRICYLQEIFSNEMAKVDDSFFHRLEESFLVEEDKKHERHPIFG
NIVDEVAYHEKYPTIYHLRKKLVDSTDKADLRLIYLALAHMIKFRGHFLIEGDLNPDNSD
VDKLFIQLVQTYNQLFEENPINASGVDAKAILSARLSKCLAEGTRIFDPVTGTTHRIEDV
VDGRKPIHVVAAAKDGTLLARPVVSWFDQGTRDVIGLRIAGGAIVWATPDHKVLTEYGWR
AAGELRKGDRVAGPGGSGNSLALSLTADQMVSALLDAEPPILYSEYDPTSPFSEASMMGL
LTNLADRELVHMINWAKRVPGFVDLTLHDQAHLLERAWLEILMIGLVWRSMEHPGKLLFA
PNLLLDRNQGKCVEGMVEIFDMLLATSSRFRMMNLQGEEFVCLKSIILLNSGVYTFLSST
LKSLEEKDHIHRALDKITDTLIHLMAKAGLTLQQQHQRLAQLLLILSHIRHMSNKRMEHL
YSMKYKNVVPLYDLLLEMLDAHRLHAGGSGASRVQAFADALDDKFLHDMLAEGLRYSVIR
EVLPTRRARTFDLEVEELHTLVAEGVVVHNCRRLENLIAQLPGEKKNGLFGNLIALSLGL
TPNFKSNFDLAEDAKLQLSKDTYDDDLDNLLAQIGDQYADLFLAAKNLSDAILLSDILRV
NTEITKAPLSASMIKRYDEHHQDLTLLKALVRQQLPEKYKEIFFDQSKNGYAGYIDGGAS
QEEFYKFIKPILEKMDGTEELLVKLNREDLLRKQRTFDNGSIPHQIHLGELHAILRRQED
FYPFLKDNREKIEKILTFRIPYYVGPLARGNSRFAWMTRKSEETITPWNFEEVVDKGASA
QSFIERMTNFDKNLPNEKVLPKHSLLYEYFTVYNELTKVKYVTEGMRKPAFLSGEQKKAI
VDLLFKTNRKVTVKQLKEDYFKKIECFDSVEISGVEDRFNASLGTYHDLLKIIKDKDFLD
NEENEDILEDIVLTLTLFEDREMIEERLKTYAHLFDDKVMKQLKRRRYTGWGRLSRKLIN
GIRDKQSGKTILDFLKSDGFANRNFMQLIHDDSLTFKEDIQKAQVSGQGDSLHEHIANLA
GSPAIKKGILQTVKVVDELVKVMGRHKPENIVIEMARENQTTQKGQKNSRERMKRIEEGI
KELGSQILKEHPVENTQLQNEKLYLYYLQNGRDMYVDQELDINRLSDYDVAAIVPQSFLK
DDSIDNKVLTRSDKARGKSDNVPSEEVVKKMKNYWRQLLNAKLITQRKFDNLTKAERGGL
SELDKAGFIKRQLVETRQITKHVAQILDSRMNTKYDENDKLIREVKVITLKSKLVSDFRK
DFQFYKVREINNYHHAHDAYLNAVVGTALIKKYPKLESEFVYGDYKVYDVRKMIAKSEQE
IGKATAKYFFYSNIMNFFKTEITLANGEIRKRPLIETNGETGEIVWDKGRDFATVRKVLS
MPQVNIVKKTEVQTGGFSKESILPKRNSDKLIARKKDWDPKKYGGFDSPTVAYSVLVVAK
VEKGKSKKLKSVKELLGITIMERSSFEKNPIDFLEAKGYKEVKKDLIIKLPKYSLFELEN
GRKRMLASAGELQKGNELALPSKYVNFLYLASHYEKLKGSPEDNEQKQLFVEQHKHYLDE
IIEQISEFSKRVILADANLDKVLSAYNKHRDKPIREQAENIIHLFTLTNLGAPAAFKYFD
TTIDRKRYTSTKEVLDATLIHQSITGLYETRIDLSQLGGDSRADPKKKRKVSPGIRRLDA
LISTSLYKKAGYKEASGSGRADALDDFDLDMLGSDALDDFDLDMLGSDALDDFDLDMLGS
DALDDFDLDMLINSRSSGSPKKKRKVGSQYLPDTDDRHRIEEKRKRTYETFKSIMKKSPF
SGPTDPRPPPRRIAVPSRSSASVPKPAPQPYPFTSSLSTINYDEFPTMVFPSGQISQASA
LAPAPPQVLPQAPAPAPAPAMVSALAQAPAPVPVLAPGPPQAVAPPAPKPTQAGEGTLSE
ALLQLQFDDEDLGALLGNSTDPAVFTDLASVDNSEFQQLLNQGIPVAPHTTEPMLMEYPE
AITRLVTGAQRPPDPAPAPLGAPGLPNGLLSGDEDFSSIADMDFSALLGSGSGSRDSREG
MFLPKPEAGSAISDVFEGREVCQPKRIRPFHPPGSPWANRPLPASLAPTPTGPVHEPVGS
LTPAPVPQPLDPAPAVTPEASHLLEDPDEETSQAVKALREMADTVIPQKEEAAICGQMDL
SHPPPRGHLDELTTTLESMTEDLNLDSPLTPELNEILDTFLNDECLLHAMHISTGLSIFD
TSLF

(**G**) Nucleotide sequence of NHEJ reporter:

Red = *mCherry*; turquoise = sgRNA target; blue = stop codon; dark red = 2-bp frameshift; green = *eGFP*

atggtgagcaagggcgaggaggataacatggccatcatcaaggagttcatgcgcttcaaggtgc
acatggagggctccgtgaacggccacgagttcgagatcgagggcgagggcgagggccgccc
ctacgagggcacccagaccgccaagctgaaggtgaccaagggtggccccctgcccttcgcctg
ggacatcctgtcccctcagttcatgtacggctccaaggcctacgtgaagcaccccgccgacatcc
ccgactacttgaagctgtccttccccgagggcttcaagtgggagcgcgtgatgaacttcgaggac
ggcggcgtggtgaccgtgacccaggactcctccctgcaggacggcgagttcatctacaaggtg
aagctgcgcggcaccaacttcccctccgacggccccgtaatgcagaagaagaccatgggctgg
gaggcctcctccgagcggatgtaccccgaggacggcgccctgaagggcgagatcaagcaga
ggctgaagctgaaggacggcggccactacgacgctgaggtcaagaccacctacaaggccaag
aagcccgtgcagctgcccggcgcctacaacgtcaacatcaagttggacatcacctcccacaacg
aggactacaccatcgtggaacagtacgaacgcgccgagggccgccactccaccggcggcatg
gacgagctgtacaagtCCGgactcagatctcgagctcaagcttcaccatggcacTAGgcca
attagccatggtgagcaagggcgaggagctgttcaccggggtggtgcccatcctggtcgagctg
gacggcgacgtaaacggccacaagttcagcgtgtccggcgagggcgagggcgatgccaccta
cggcaagctgaccctgaagttcatctgcaccaccggcaagctgcccgtgccctggcccaccctc
gtgaccaccctgacctacggcgtgcagtgcttcagccgctaccccgaccacatgaagcagcac
gacttcttcaagtccgccatgcccgaaggctacgtccaggagcgcaccatcttcttcaaggacga
cggcaactacaagacccgcgccgaggtgaagttcgagggcgacaccctggtgaaccgcatcg
agctgaagggcatcgacttcaaggaggacggcaacatcctggggcacaagctggagtacaact
acaacagccacaacgtctatatcatggccgacaagcagaagaacggcatcaaggtgaacttcaa
gatccgccacaacatcgaggacggcagcgtgcagctcgccgaccactaccagcagaacaccc
ccatcggcgacggccccgtgctgctgcccgacaaccactacctgagcacccagtccgccctga
gcaaagaccccaacgagaagcgcgatcacatggtcctgctggagttcgtgaccgccgccggg
atcactcTcggcatggacgagctgtacaagatgtacccatacgatgttccagattacgctTAA

Supplementary Table 1. Oligonucleotide sequences used in this study. The sequences of the sgRNAs were designed according to the protocol described in Ran *et al*[1].

Gene target	sgRNA name	Oligos
NHEJ reporter	sgMCE	sgMCE-fwd<br>5'-caccTTGAGCTCGAGATCTGAGTC-3'<br>sgMCE-rev<br>5'-aaacGACTCAGATCTCGAGCTCAA-3'
GAL4-UAS	sgGAL	sgGAL-fwd<br>5'-caccGAGCACTGTCCTCCGAACGT-3'<br>sgGAL-rev<br>5'-aaacACGTTCGGAGGACAGTGCTC-3'
RBM20	sgRBM	sgRBM-fwd<br>5'-caccGGTCTCGTAGTCCGGTGAGC-3'<br>sgRBM-rev<br>5'-aaacGCTCACCGGACTACGAGACC-3'
PHOX2B	sgPHOX	sgPHOX-fwd<br>5'-caccCCTACGAGTCCTGTATGGCT-3'<br>sgPHOX-rev<br>5'-aaacAGCCATACAGGACTCGTAGG-3'
EMX1	sgEMX	sgEMX-fwd<br>5'-caccGAGTCCGAGCAGAAGAAGAA-3'<br>sgEMX-rev<br>5'-aaacTTCTTCTTCTGCTCGGACTC-3'
POU5F1	sgPOU5F1-3	sgPOU5F1-3-fwd<br>5'-caccGTGGGGGGAGAAACTGAGGCGA-3'<br>sgPOU5F1-3-rev<br>5'-aaacTCGCCTCAGTTTCTCCCCCCAC-3'
POU5F1	sgPOU5F1-5	sgPOU5F1-5-fwd<br>5'-caccGGATGTTTGCCTAATGGTGG-3'<br>sgPOU5F1-5-rev<br>5'-aaacCCACCATTAGGCAAACATCC-3'
POU5F1	sg-Sa-POU5F1	sg-Sa-POU5F1-fwd<br>5'-caccGGTGGGGGGAGAAACTGAGGCG-3'<br>sg-Sa-POU5F1-rev<br>5'-aaacCGCCTCAGTTTCTCCCCCCACC-3'

Supplementary Methods

Immunofluorescent staining. HEK 293T cells were plated at approximately 0.5×10^6 cells/well in 35-mm dishes (Mattek) and cultured overnight at 37°C under 5% CO_2 before transfection. To express ERT-**A**-VPR, the cells were co-transfected with plasmids encoding each protein fragment at 1:1 ratio using TransIT-293 (Mirus Bio) following the manufacturer's procedure. The growth medium was replaced with fresh DMEM medium with 10% FBS, 4OHT (10 μM) and/or rapamycin (10 nM) 24 h after transfection. The cells were washed with PBS and fixed with fixing solution (4% formaldehyde/0.1% Triton-X/PBS) for 15 min at room temperature. The cells were washed with PBS and incubated in blocking buffer (3% bovine serum albumin/0.1 % Triton-X/PBS) for 1 h at room temperature. The blocking solution was aspirated, and the cells were incubated with α-HA rabbit monoclonal antibody (Cell Signaling, C29F4 #3724) and α-myc mouse monoclonal antibody (Cell Signaling, 9B11 #2276) diluted at 1:1000 ratio in antibody buffer (3% bovine serum albumin/0.1 % Triton-X/PBS) overnight at 4°C. The cells were washed with PBS and incubated with Alexa Fluor 488 conjugated α-mouse IgG antibody (Invitrogen #A11029, 1:1000 dilution), Alexa Fluor 647 conjugated goat α-rabbit IgG antibody (Invitrogen #A21245, 1:1000 dilution), and Hoescht 33342 (Invitrogen H3570, 2 μg/mL) diluted in antibody buffer for 1 h at room temperature. The cells were subsequently washed with PBS and imaged using a Zeiss LSM 510 META Confocal Laser-Scanning microscope.

For expression of ERT-fused full-length constructs, the cells were co-transfected with a plasmid encoding ERT-dCas9-VPR or ERT-dCas9-VPR$^{\Delta NLS}$, and another plasmid encoding sgGAL at 1:1 ratio. The cells were induced and fixed in the same procedure as above. The cells were stained overnight at 4°C using α-HA rabbit antibody for ERT-dCas9-VPR detection or α-myc mouse antibody for ERT-dCas9-VPR$^{\Delta NLS}$ detection. The cells were subsequently incubated with Alexa Fluor 647 conjugated goat α-mouse IgG antibody (Invitrogen #A21236, 1:1000 dilution) or Alexa Fluor 647 conjugated goat α-rabbit IgG antibody (Invitrogen #A21245, 1:1000 dilution), and Hoescht 33342 (Invitrogen H3570, 2 μg/mL) diluted in antibody buffer for 2 h at room temperature. The cells were subsequently washed with PBS and imaged using a Zeiss Axio Observer Z1 inverted fluorescence microscope.

Supplementary Reference

1. Ran, F. A. *et al*. Genome engineering using the CRISPR-Cas9 system. *Nat Protoc*. **8,** 2281–2308 (2013).
